# Neuronal SIRT1 regulates macronutrient-based diet selection through FGF21 and oxytocin signalling in mice

**DOI:** 10.1038/s41467-018-07033-z

**Published:** 2018-11-02

**Authors:** Sho Matsui, Tsutomu Sasaki, Daisuke Kohno, Keisuke Yaku, Ayumu Inutsuka, Hiromi Yokota-Hashimoto, Osamu Kikuchi, Takayoshi Suga, Masaki Kobayashi, Akihiro Yamanaka, Akihiro Harada, Takashi Nakagawa, Tatsushi Onaka, Tadahiro Kitamura

**Affiliations:** 1 Laboratory of Metabolic Signal, Metabolic Signal Research Center, Institute for Molecular and Cellular Regulation, Gunma University, 3-39-15 Showa-machi, Maebashi, Gunma 371-8512 Japan; 2 Advanced Scientific Research Leaders Development Unit, Gunma University, 3-39-15 Showa-machi, Maebashi, Gunma 371-8512 Japan; 3 Frontier Research Core for Life Science, University of Toyama, 2630 Sugitani, Toyama, Toyama, 930-0194 Japan; 4 Department of Metabolism and Nutrition, Graduate School of Medicine and Pharmaceutical Science for Research, University of Toyama, 2630 Sugitani, Toyama, Toyama, 930-0194 Japan; 5 Department of Neuroscience II, Research Institute of Environmental Medicine, Nagoya University, Furocho, Nagoya, 464-8601 Japan; 6 Division of Brain and Neurophysiology, Department of Physiology, Jichi Medical University, 3311-1 Yakushiji, Shimotsuke, Tochigi 329-0498 Japan; 7 Department of Cell Biology, Graduate School of Medicine, Osaka University, 2-2 Yamadaoka, Suita, Osaka, 565-0871 Japan

## Abstract

Diet affects health through ingested calories and macronutrients, and macronutrient balance affects health span. The mechanisms regulating macronutrient-based diet choices are poorly understood. Previous studies had shown that NAD-dependent deacetylase sirtuin-1 (SIRT1) in part influences the health-promoting effects of caloric restriction by boosting fat use in peripheral tissues. Here, we show that neuronal SIRT1 shifts diet choice from sucrose to fat in mice, matching the peripheral metabolic shift. SIRT1-mediated suppression of simple sugar preference requires oxytocin signalling, and SIRT1 in oxytocin neurons drives this effect. The hepatokine FGF21 acts as an endocrine signal to oxytocin neurons, promoting neuronal activation and *Oxt* transcription and suppressing the simple sugar preference. SIRT1 promotes FGF21 signalling in oxytocin neurons and stimulates *Oxt* transcription through NRF2. Thus, neuronal SIRT1 contributes to the homeostatic regulation of macronutrient-based diet selection in mice.

## Abstract (web summary)

SIRT1 is a NAD^+^-dependent deacetylase whose functions have been linked to organismal longevity, aging and metabolism. Here, Matsui and colleagues show that neuronal SIRT1 can affect nutrient-related dietary choice in mice, and this effect is mediated by FGF21 signalling and oxytocin.

## Introduction

Diabetes and obesity are the third and fourth leading health risk factors, respectively, on a global scale^1^. Lifestyle modification, including diet therapy, comprises an important intervention against diabetes and obesity, but low adherence hinders its efficacy. The efficacy of diet therapy depends on both the quantity (caloric intake) and quality (macronutrient balance) of the ingested diet.

A large prospective epidemiological cohort study showed that excessive dietary carbohydrates impair health span and promote mortality and that replacing intake of carbohydrates with fat reduces mortality^2^. However, another study in overweight and obese men reported that replacing a high-carbohydrate baseline diet with an isocaloric low-carbohydrate ketogenic diet was not accompanied by increased body fat loss but it was associated with relatively small increases in energy expenditure^3^. Despite long-standing debates on the health benefits of fat restriction versus carbohydrate restriction^4,5^, both fat and carbohydrates (particularly simple sugars) are deemed highly rewarding. However, macronutrient selection behaviour cannot be fully understood by studying only hedonic feeding. For example, the central melanocortin system, which plays a major role in regulating caloric intake homeostatically, is implicated in the regulation of macronutrient preferences^6–8^, and some neuropeptides and endocrine signals regulate intake of specific macronutrients^9^. Nevertheless, an integrated understanding of the mechanisms that regulate macronutrient-based diet selection is lacking, yet it is required for developing a diet therapy that facilitates adherence.

To elucidate the homoeostatic regulation of macronutrient selection, we focused on the NAD^+^-dependent deacetylase, SIRT1. SIRT1 plays a dual role in controlling energy homoeostasis: SIRT1 in peripheral tissues promotes the use of fat as substrate, and SIRT1 in the central nervous system (CNS) promotes homoeostatic feeding control by improving hormone sensing^10,11^. Therefore, we hypothesized that CNS SIRT1 might regulate macronutrient intake by shifting the macronutrient preference to match the metabolic need in peripheral tissues (i.e. supply the appropriate substrate for use). SIRT1 is also important for prolonging lifespan in multiple organisms through a diet regimen called caloric restriction^11^. Across species, caloric restriction causes shifts in survival priorities from reproduction to self-maintenance, and simultaneously, this shift reprograms the metabolism from using carbohydrate to using fat^12^. This metabolic reprogramming is consistent with the priority shift to self-maintenance, because glycolysis, which occurs at a high rate during carbohydrate use, is the metabolism of choice for replicating cells, including stem cells and cancer cells^13,14^. Therefore, our hypothesis is consistent with the evolutionarily-conserved principle that caloric restriction will shift metabolism to match the shift in survival priority.

By testing the hypothesis, here we show that neuronal SIRT1 shifts diet choice from sucrose to fat in mice, matching the peripheral metabolic shift. Mechanistically, neuronal SIRT1 regulates simple sugar preference through FGF21 and oxytocin signalling. Thus, neuronal SIRT1 contributes to the homoeostatic regulation of macronutrient-based diet selection in mice.

## Results

### Neuronal SIRT1 regulates macronutrient-based diet selection

**Fig. 1:**
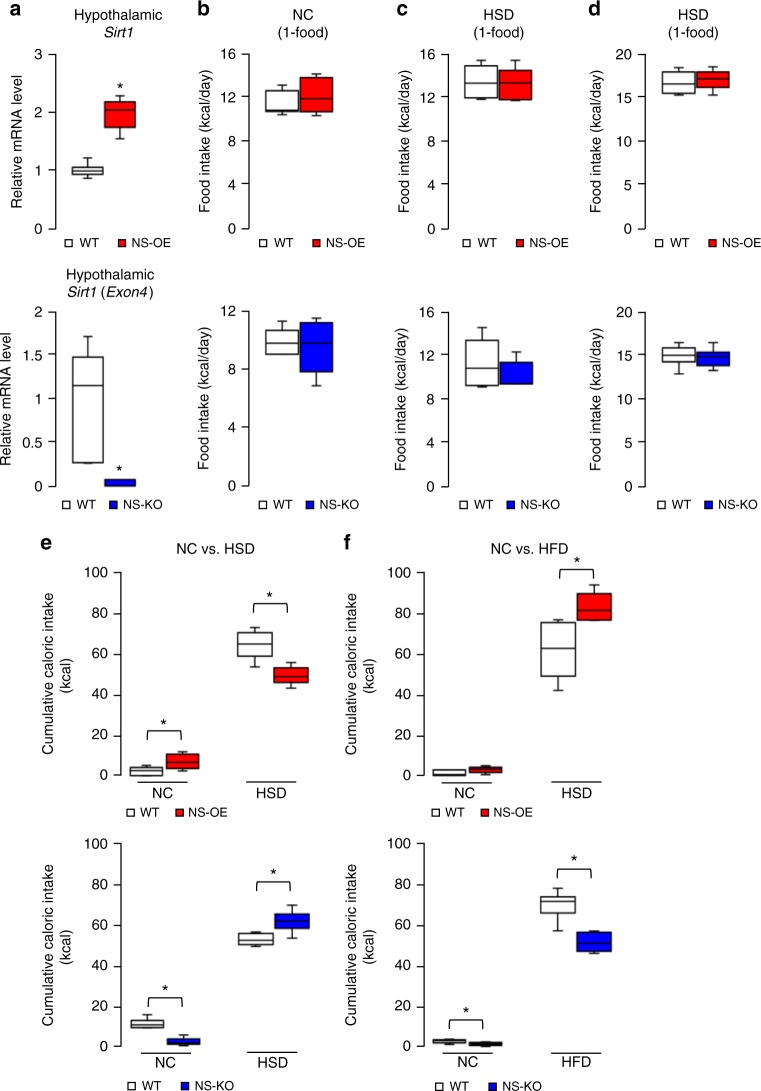
Neuronal SIRT1 regulates macronutrient-based diet selection. **a** Hypothalamic *Sirt1* mRNA expression of NS-OE (red) and NS-KO (blue) mice compared to wild-type mice (WT) (*n* = 6 per group). **b**–**d** Food intake (kcal/d) for NS-OE and NS-KO mice fed NC only (**b**), HSD only (**c**) and HFD only (**d**) (*n* = 6 per group). **e**, **f** Cumulative caloric intake for two-choice diets in 5-day diet selection experiments: NC vs. HSD (**e**) and NC vs. HFD (**f**) (*n* = 6 per group). Data are shown as box and whisker plots (centre line, median; box limits, upper and lower quartiles; whiskers, the minimum and maximum value of a data set). *p < 0.05. See also Supplementary Fig. 1

To test whether central SIRT1 could simultaneously promote fat preference and suppress carbohydrate preference, we generated neuron-specific SIRT1 overexpression (NS-OE) and neuron-specific SIRT1 knockout (NS-KO) mice as neuron-specific SIRT1 gain-of-function and loss-of-function models, respectively. Briefly, neuron-specific *Tau-Cre* mice^15^ were crossed with either *Rosa26-Sirt1* mice^10^ or *Sirt1-flox* mice^16^ to generate NS-OE and NS-KO mice, respectively. We confirmed that hypothalamic *Sirt1* expression levels in NS-OE and NS-KO mice were twofold and negligible, respectively, compared to those in wild-type littermates (Fig. 1a). The acetylation levels of SIRT1 substrates (FOXO1^17^, p53^18,19^ and NF-κB^20^) in mice that received 3rd intracerebroventricular (ICV) injection of trichostatin A (TSA), an histone deacetylase inhibitor, were decreased in the hypothalamus of NS-OE mice (Supplementary Fig. 1a), confirming that NS-OE mice served as a SIRT1 gain-of-function model. Both modified mouse strains were viable, and food intake and body weight were not different from wild-type mice when fed normal chow (NC), a high-sucrose diet (HSD) or a high-fat diet (HFD), without a choice in diet (Fig. 1b–d; Supplementary Fig. 1b).

When NS-OE and NS-KO mice were given diet choices (NC vs. HSD or NC vs. HFD), they showed different preferences. The preference for the HSD was reduced in NS-OE and increased in NS-KO mice (Fig. 1e). Conversely, the preference for HFD was increased in NS-OE and reduced in NS-KO mice (Fig. 1f). In 5-day experiments, these preferences resulted in significant differences in weight gain when mice selected between NC and HFD but not when they selected between NC and HSD (Supplementary Fig. 1c, d). Therefore, neuronal SIRT1 promoted fat preference and suppressed sucrose preference.

### Neuronal SIRT1 regulates sucrose preference through oxytocin

**Fig. 2:**
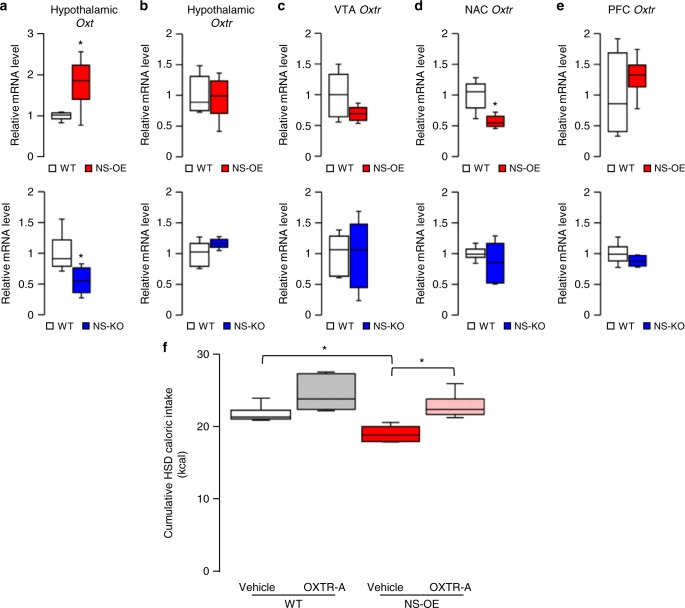
Neuronal SIRT1 regulates sucrose preference through Oxt. **a**–**e** Relative RNA expression levels, for NS-OE (red) and NS-KO (blue) mice; *Oxt* expression in the hypothalamus (**a**); *Oxtr* expression in the hypothalamus (**b**), ventral tegmental (VTA) (**c**), nucleus accumbens (NAc) (**d**) and prefrontal cortex (PFC) (**e**) (*n* = 6 per group). **f** The effect of an IP injection of Oxt receptor antagonist OXTR-A (L-368,899) on HSD intake in NS-OE and littermate control mice (*n* = 5 per group). Data are shown as box and whisker plots (centre line, median; box limits, upper and lower quartiles; whiskers, the minimum and maximum value of a data set). *p < 0.05. See also Supplementary Fig. 2

If the general alteration in the hedonic system were the primary cause of the altered preferences for fat and sucrose in these modified mouse strains, then the preferences for both fat and sucrose would change in the same direction (instead of opposite directions), because they are both palatable to mice and drive reward circuits in the brain^21^. Therefore, we investigated the expression of genes known to encode neuropeptides and receptors that selectively regulate preference for a particular macronutrient^9^, such as neuropeptide Y (*Npy*), oxytocin (*Oxt*), galanin (*Gal*) and neuromedin U (*Nmu*). We screened samples of the hypothalamus, ventral tegmental area (VTA), nucleus accumbens (NAc) and prefrontal cortex (PFC) from NS-KO and NS-OE mice. Hypothalamic expression of *Oxt*, which selectively suppresses the preference for carbohydrates but not fat^22–27^, was positively correlated with *Sirt1* expression (Fig. 2a). SIRT1, however, did not affect the hypothalamic expression of *Npy*, NPY receptors Y1 and Y5 (*Y1R* and *Y5R*), *Gal* and *Nmu* (Supplementary Fig. 2a–e).

Next, we assessed the expression of Oxt receptor (*Oxtr*) in the hypothalamus, VTA, NAc and PFC of wild-type, NS-OE and NS-KO mice. *Oxtr* expression levels were not significantly different between wild-type mice and NS-KO mice in any of these areas or between wild-type and NS-OE mice in any area except the NAc (Fig. 2b–e). To examine whether Oxt action on its receptor is necessary for the effect of neuronal SIRT1 on HSD preference, we blocked OXTRs using intraperitoneal (IP) injections of the OXTR antagonist L-368,899 (OXTR-A), which can penetrate the blood–brain barrier. OXTR-A treatment abolished the suppression of HSD intake in NS-OE mice (Fig. 2f). This result indicated that the action of Oxt on OXTR is required for neuronal SIRT1 to suppress the sucrose preference.

### SIRT1 in Oxt neurons regulates preference for sucrose

**Fig. 3:**
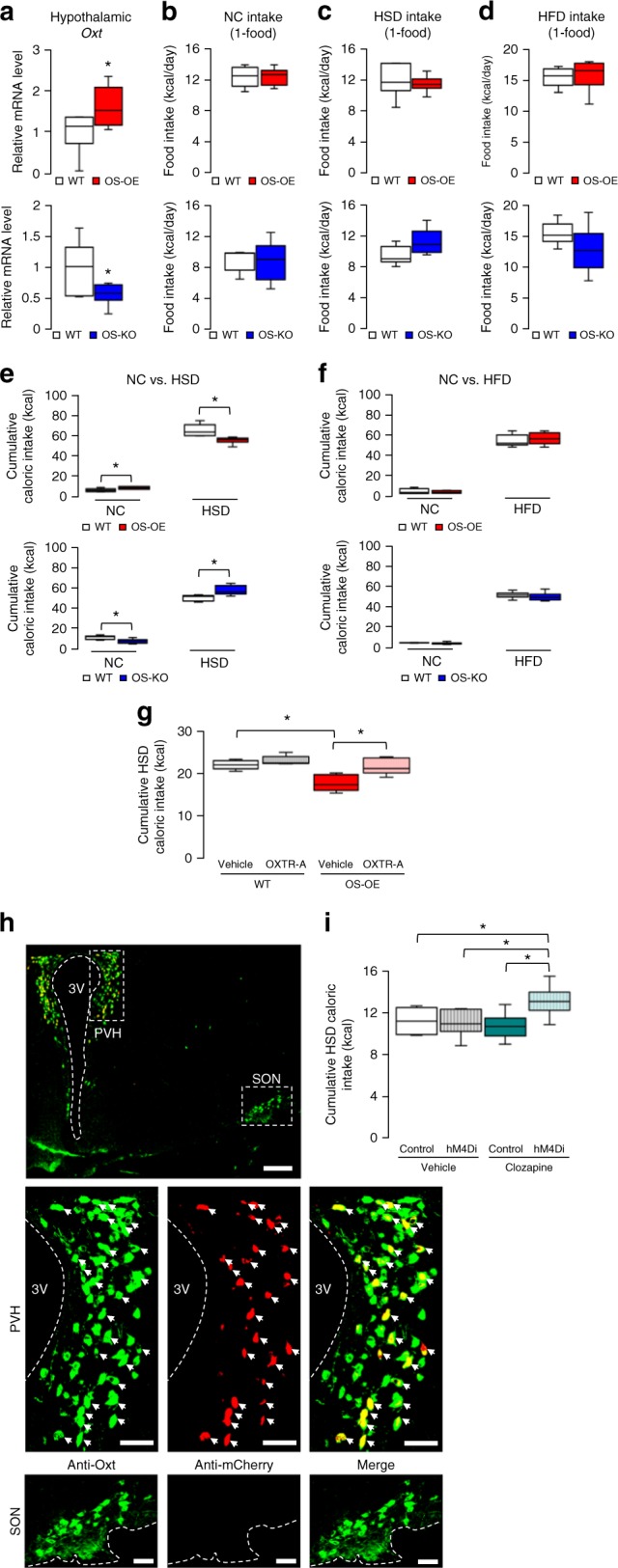
SIRT1 in Oxt neurons regulates sucrose preference. **a** Hypothalamic *Oxt* mRNA expression in WT, OS-OE (red) and OS-KO (blue) mice (*n* = 6 per group). **b**–**d** Food intake (kcal/d) for OS-OE and OS-KO mice fed NC only (**b**), HSD only (**c**) and HFD only (**d**) (*n* = 6 per group). **e**, **f** Cumulative caloric intake for two-choice diets in 5-day diet selection experiments: NC vs. HSD (**e**) and NC vs. HFD (**f**) (*n* = 6 per group). **g** The effect of an IP injection of the Oxt receptor antagonist OXTR-A (L-368,899) on HSD intake in OS-OE and littermate control (WT) mice (*n* = 6 per group). **h** Representative immunofluorescent images of the paraventricular nucleus (PVH) and the supraoptic nucleus (SON) of the hypothalamus of OS-OE mice used in the DREADD experiments. Co-localization of Oxt (green) and mCherry (red) signals is seen in the merged image (white arrows). The size of the scale bar is 200 μm for the low magnification panel and 50 μm for the high magnification panels. **i** Clozapine-induced inhibition of Oxt neuronal activity by hM4Di increased HSD intake in OS-OE mice. The changes in HSD intake (clozapine-vehicle) during the course of 3-day NC vs. HSD feeding (*n* = 8 per group). Data are shown as box and whisker plots (centre line, median; box limits, upper and lower quartiles; whiskers, the minimum and maximum value of a data set). *p < 0.05. 3V, 3rd ventricle. See also Supplementary Figs. 3 and 4

Expression of *Oxt* positively correlated with that of *Sirt1* in NS-OE and NS-KO mice (Figs. 1a and 2a), which suggested that SIRT1 in Oxt neurons may regulate *Oxt* expression. Therefore, we next tested whether SIRT1 in Oxt neurons was sufficient to regulate sucrose preference. We genetically manipulated *Sirt1* expression specifically in Oxt neurons by generating Oxt-neuron-specific SIRT1 overexpression (OS-OE) and Oxt-neuron-specific SIRT1 knockout (OS-KO) mice. Briefly, Oxt-neuron-specific *Oxt-ires-Cre* mice^28^ were crossed with either *Rosa26-Sirt1* mice or *Sirt1-flox* mice to generate OS-OE and OS-KO mice, respectively. We performed immunohistochemistry to confirm that SIRT1 expression was manipulated specifically in Oxt neurons, and that the acetylation levels of SIRT1 substrates were decreased specifically in Oxt neurons of OS-OE mice that received 3rd ICV injection of TSA (Supplementary Fig. 3). We found that hypothalamic *Oxt* expression levels were increased and decreased in OS-OE and OS-KO mice, respectively, compared to those in wild-type littermates (Fig. 3a). Therefore, SIRT1 in Oxt neurons controlled *Oxt* expression.

In the absence of a dietary choice, the food intake and body weight of OS-OE and OS-KO mice were not significantly different from those of wild-type mice (Fig. 3b–d; Supplementary Fig. 4a). Moreover, the locomotor activity, metabolic rates and plasma leptin levels of these mice were not significantly different from those of control mice (Supplementary Fig. 4e–j). On the other hand, when offered a choice between NC and HSD, the HSD preference was reduced in OS-OE mice and elevated in OS-KO mice compared to wild-type mice, with similar results for males and females (Fig. 3e; Supplementary Fig. 4d). These findings were consistent with altered sucrose preferences observed in NS-OE and NS-KO mice under the diet-choice settings. Consequently, body weight was reduced and tended to be elevated in these groups, respectively, compared to wild-type mice over the 5-day experimental period (Supplementary Fig. 4b). Meanwhile, when offered a choice between NC and HFD, food intake and subsequent weight gain were not different between wild-type and either OS-OE or OS-KO mice (Fig. 3f; Supplementary Fig. 4c), which is consistent with the carbohydrate-specific effect of Oxt on preference. Therefore, SIRT1 in Oxt neurons regulated preference for sucrose, but not fat, in these mice.

Next, we tested the necessity of the Oxt action on its receptor by OXTR antagonist. We found that OXTR-A treatment abolished the reduced HSD intake in OS-OE mice (Fig. 3g). We then tested the necessity of the neural activation of Oxt neurons for the reduced HSD intake in OS-OE mice by using an inhibitory designer receptor exclusively activated by designer drugs (DREADDs)^29^ in Oxt neurons in the paraventricular nucleus of the hypothalamus (PVH). The inhibitory DREADD receptor hM4Di, when stimulated by the DREADD ligand clozapine, activates G-protein inwardly rectifying potassium channels, thereby hyperpolarizing and attenuating neuronal activity^29^. We infected AAV2-hSyn-FLEX-mCherry (control) or AAV2-hSyn-FLEX-hM4Di-mCherry (hM4Di) in PVH Oxt neurons of OS-OE mice. These mice have the *Oxt-ires-Cre* allele, and recombination of the FLEX vectors by the Cre recombinase would be expected to induce expression of the target genes (mCherry or hM4Di-mCherry) only in *Cre*-positive PVH Oxt neurons. We confirmed that the control or hM4Di was selectively expressed in Oxt neurons located in the PVH, but not in the supraoptic nucleus (SON), based on the co-localization of signals emitted from anti-mCherry stains and anti-Oxt stains (Fig. 3h). We injected IP clozapine (1 μg/kg) or vehicle (phosphate-buffered saline (PBS); pH 7.4) in OS-OE mice expressing control or hM4Di. Injection of clozapine into hM4Di-expressing OS-OE mice increased HSD intake compared to that of vehicle injection (Fig. 3i). On the other hand, clozapine injection to control-expressing OS-OE mice did not significantly alter HSD intake as compared to that of vehicle injection (Fig. 3i). Therefore, activation of Oxt neurons mediates the effect of SIRT1 in suppressing sucrose preference in OS-OE mice. These results collectively indicated that SIRT1 in Oxt neurons is sufficient to suppress the sucrose preference and that the effect of SIRT1 on sucrose preference is mediated by released Oxt upon neural activation and its action on OXTR.

### SIRT1 in Oxt neurons regulates simple sugar preference

**Fig. 4:**
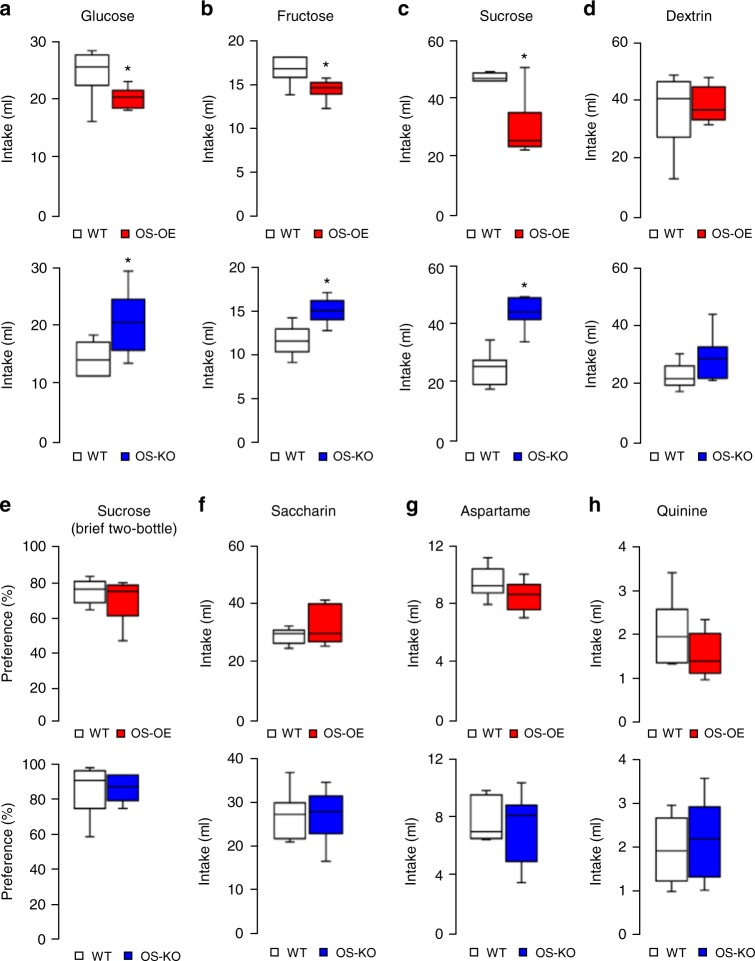
SIRT1 in Oxt neurons specifically regulates preferences for simple sugars. Two-bottle choice tests in OS-OE (red) and OS-KO (blue) mice. **a**–**d** Results of 3-day two-bottle choice tests (100 mM glucose (**a**), 100 mM fructose (**b**), 100 mM sucrose (**c**) and 2% dextrin (**d**)) (*n* = 7 in the WT group; *n* = 6 in the OS-OE and OS-KO groups). **e** Results of 10-min brief access two-bottle choice tests for 100 mM sucrose over water (*n* = 6 per group). **f**, **g** Results of two-bottle choice tests with artificial sweeteners (0.2% saccharin (**f**) and 0.2% aspartame (**g**)) over water, given for 3 days in sugar-naive mice (*n* = 8 per group). **h** Results of 3-day two-bottle choice tests with 1.5 mM quinine (bitter taste) (*n* = 7 in the WT group; *n* = 6 in the OS-OE and OS-KO groups). Data are shown as box and whisker plots (centre line, median; box limits, upper and lower quartiles; whiskers, the minimum and maximum value of a data set). *p < 0.05. See also Supplementary Fig. 9

We next asked if the preference alteration in OS-OE and OS-KO mice was specific to sucrose or if the phenomenon could be generalized to all carbohydrates. We addressed these questions by analysing the preference phenotypes of OS-OE and OS-KO mice relative to various carbohydrates using two-bottle choice tests over 3 days. These experiments revealed that the preference phenotype was specific for simple sugars (glucose, fructose and sucrose) (Fig. 4a–c), and not for complex sugars (dextrin) (Fig. 4d).

Preference for simple sugars could be mediated by orosensory stimuli such as taste and by a post-ingestive metabolic effect of sugars. To address the effect of taste versus the post-ingestive metabolic effect of sugars, we performed brief two-bottle choice tests over 10 min, which minimized the post-ingestive metabolic effect. However, when we conducted these brief tests with sucrose, both OS-OE and OS-KO mice showed no significant differences from wild-type mice (Fig. 4e). We next tested the artificial sweeteners, saccharin and aspartame, in sugar-naive mice. We used sugar-naive mice to avoid the potential post-ingestive metabolic effect of non-nutritive artificial sweetener induced by conditioning-based learning^30,31^. Two-bottle choice tests with these sweeteners revealed that preferences to the sweeteners were not different in OS-OE mice and OS-KO mice in the 3-day tests (Fig. 4f, g). There was no significant difference in the preference for bitter-tasting quinine in these mice (Fig. 4h). These data collectively indicated that SIRT1 regulated the preference only for simple sugars, and that the taste effect of sugar did not contribute to the SIRT1 regulation of the simple sugar preference. Therefore, the metabolic effects of sugars played predominant roles in the SIRT1 regulation of the simple sugar preference.

### Oxt neurons in the PVH respond to FGF21

If SIRT1 regulates sucrose preference through Oxt and the effect is mediated primarily by the post-ingestive metabolic effect of sugars, metabolic signals that represent simple sugar ingestion may activate Oxt neurons. FGF21 is secreted from the liver upon simple sugar ingestion in rodents and humans^32,33^, and it suppresses simple sugar intake and preference by signalling to the PVH^32,34^. The PVH harbours numerous subtypes of peptidergic neurons, including Oxt neurons^35^. The expression of the neural activation marker, c-Fos, in PVH Oxt neurons was induced by oral ingestion of sucrose^27^ and intra-gastric ingestion of sweetened condensed milk^36^. Therefore, we hypothesized that FGF21 secreted upon simple sugar ingestion may be the metabolic signal to Oxt neurons, which control simple sugar preference by way of negative feedback.

**Fig. 5:**
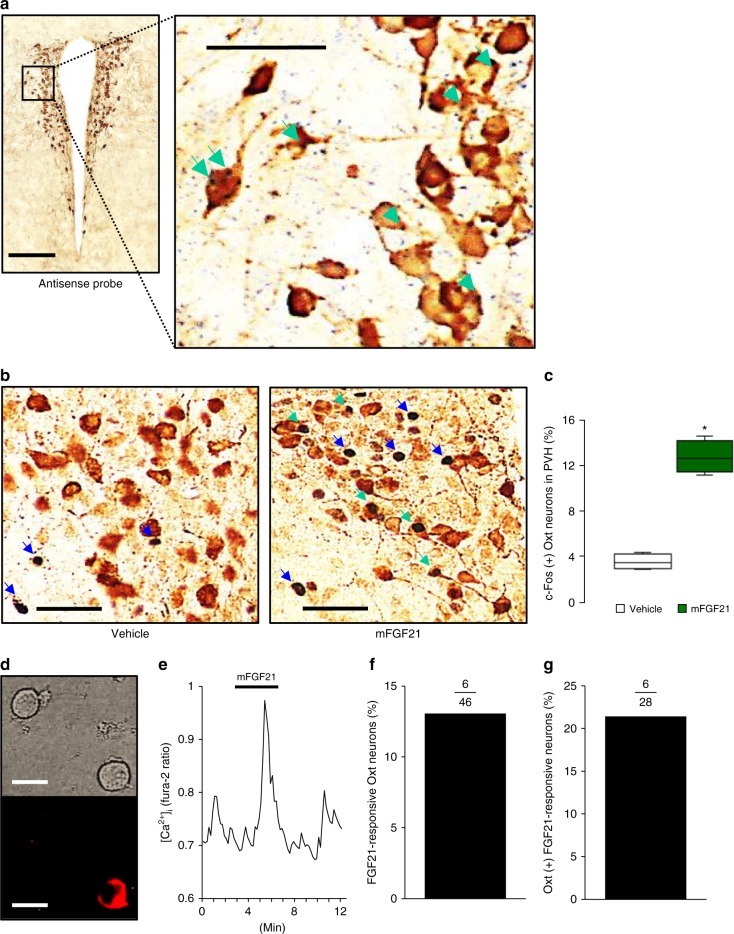
Oxt neurons in the PVH respond to FGF21. **a** Photomicrographs depict coronal PVH sections from wild-type C57BL/6 mice. Sections were subjected to in situ hybridization to identify *β-Klotho* mRNA (*Klb*, blue dots). Then, sections were immunostained to identify Oxt protein (brown) expression. Green arrows indicate *Klb* mRNA staining in Oxt neurons. **b** Photomicrographs depict coronal PVH sections from wild-type C57BL/6 mice injected with murine FGF21 (mFGF21) (1 mg/kg, IP) or water (vehicle). The sections were immunostained for c-Fos (black) and Oxt (brown). Blue arrows indicate Oxt (−) c-Fos (+) neurons; green arrows indicate c-Fos (+) Oxt (+) neurons. **c** The percentage of c-Fos (+) cells among PVH Oxt neurons after an IP injection of vehicle or mFGF21 (*n* = 4 per group). **d**, **e** A representative [Ca^2+^]_i_ result, expressed by fura-2 fluorescence ratio (F340/F380), in an ex vivo single-cell Oxt-neuron. Addition of 50 ng/ml mFGF21 for 5 min to perfusate (KRB containing 10 mM glucose) increased [Ca^2+^]_i_ in a single PVH neuron (**e**), which was subsequently shown to be IF to Oxt by immunocytochemical staining (**d**). **f**, **g** Percentage of mFGF21-responding Oxt neurons among PVH Oxt neurons (**f**) and mFGF21-responding PVH neurons (**g**). The number above the bar indicates the number of neurons that responded over number examined. The length of scale bar is 200 μm for the low magnification panel and 50 μm for the high magnification panel in **a**; 50 μm in **b**; and 20 μm in **d**. Data are shown as box and whisker plots (centre line, median; box limits, upper and lower quartiles; whiskers, the minimum and maximum value of a data set). *p < 0.05. See also Supplementary Fig. 5

For FGF21 to work as a metabolic feedback signal to Oxt neurons, these cells must express FGF21 receptors. FGF21 receptor is composed of β-Klotho (*Klb*) and the FGF receptor heterodimer. The distribution of FGF21 receptors is defined by the restricted expression pattern of β-Klotho because FGF receptors are expressed ubiquitously^37^. Therefore, we checked for the co-localization of *Klb* and *Oxt* expression by performing in situ hybridization and immunohistochemistry on PVH sections. We detected *Klb* mRNA staining in some Oxt-immunoreactive neurons (Fig. 5a; Supplementary Fig. 5a). Oxt neurons are also present in the SON, and we also detected *Klb* mRNA staining in some Oxt-immunoreactive SON neurons (Supplementary Fig. 5b). We next tested whether FGF21 could activate Oxt neurons in vivo. An IP injection of murine FGF21 (mFGF21) induced c-Fos expression in 13% of the PVH Oxt neurons (Fig. 5b, c), including both magnocellular and parvocellular Oxt neurons. On the other hand, mFGF21 did not significantly induce c-Fos expression in SON Oxt neurons (Supplementary Fig. 5c–d). Therefore, a fraction of Oxt neurons represented one of the targets of FGF21 in the PVH.

Neuronal activation by a hormone in vivo, however, can be triggered through the direct action of the hormone on the target neuron and also by indirect action through neural circuits. Therefore, we examined whether FGF21 directly activates PVH Oxt neurons using ex vivo calcium imaging of the dispersed single-cell PVH neurons, so that the contribution of neural circuits to neural activation was eliminated. Treatment of mFGF21 (50 ng/ml) increased intracellular calcium concentration ([Ca^2+^]_i_) in the PVH neurons that were subsequently shown to be immunofluorescent (IF) to Oxt (Fig. 5d, e). Of 46 PVH Oxt neurons, 6 of them (13%) showed increased [Ca^2+^]_i_ in response to mFGF21 (Fig. 5f), while 6 out of 28 (21%) mFGF21-activated PVH neurons were Oxt-positive (Fig. 5g). These results showed that FGF21 can directly activate some PVH Oxt neurons ex vivo.

This evidence collectively suggested that FGF21 stimulates Oxt neuronal activity linked to Oxt release at axon terminals. Although Oxt is also released from Oxt neuronal somata and dendrites independent of an action potential^38^, FGF21 failed to induce significant somato-dendritic release of Oxt (Supplementary Fig. 5e). Therefore, we speculate that FGF21 stimulates Oxt neurons and that release of Oxt at projection site(s) mediates the feedback suppression of simple sugar preference by FGF21.

### FGF21 regulates Oxt expression via AKT

**Fig. 6:**
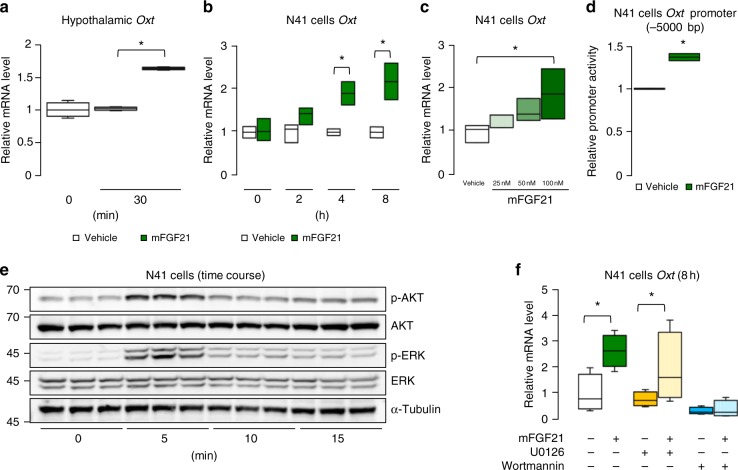
FGF21 stimulates *Oxt* expression through AKT. **a** Hypothalamic *Oxt* mRNA expression levels after IP injection of mFGF21 (1 mg/kg) or water (vehicle) (*n* = 4 per group). **b**, **c** The relative expression of *Oxt* mRNA in hypothalamic N41 cells after mFGF21 treatment: time-course data (**b**) and dose-response data (**c**) (*n* = 3 per group). **d** Luciferase activity of *Oxt* promoter of −5000 bp in N41 cells was measured in response to 100 nM mFGF21 and vehicle (water) (*n* = 3 per group). **e** Western blot analyses of N41 lysates after 100 nM mFGF21 treatment for 0, 5, 10 or 15 min (*n* = 3 per time point). **f** Effect of inhibiting ERK signalling or AKT signalling on FGF21-induced *Oxt* expression. N41 cells underwent 30-min pre-treatment with or without the ERK inhibitor (10 μM U0126) or the PI3K inhibitor (10 μM wortmannin); subsequently, cells were incubated for 8 h in the absence or presence of 100 nM mFGF21 (*n* = 4 per group). Data are shown as box and whisker plots (centre line, median; box limits, upper and lower quartiles; whiskers, the minimum and maximum value of a data set). *p < 0.05. Data in **a**, **b**, **c**, **d**, **f** were replicated more than three times, and data in **e** were replicated twice in the laboratory

Because sucrose ingestion also induces Oxt expression as well as Oxt neural activation^27^, we next analysed the effects of FGF21 on *Oxt* expression in vivo and in vitro. An IP injection of mFGF21 induced *Oxt* expression in the mouse hypothalamus (Fig. 6a). When cells from the mouse hypothalamic N41 cell line were treated with mFGF21, *Oxt* expression and *Oxt* promoter (−5000 bp) activity were also induced (Fig. 6b–d). Binding of FGF21 to its receptor (KLB/FGFR heterodimer) activates ERK and AKT signalling^37^, and these signalling pathways were also activated by mFGF21 in N41 cells (Fig. 6e). FGF21-induced *Oxt* expression was blocked by adding wortmannin (a PI3K inhibitor, which blocks subsequent AKT activation), but not by UO126 (an ERK1/2 inhibitor) (Fig. 6f). Therefore, FGF21-dependent Oxt expression is mediated by FGF21-KLB-AKT signalling. Because ERK phosphorylation is used as a marker of neural activation, FGF21 stimulated both neural activation and Oxt expression in Oxt neurons through these two kinases that act downstream of the FGF21 receptor.

### SIRT1 promotes Oxt expression via FGF21-KLB-AKT signalling

**Fig. 7:**
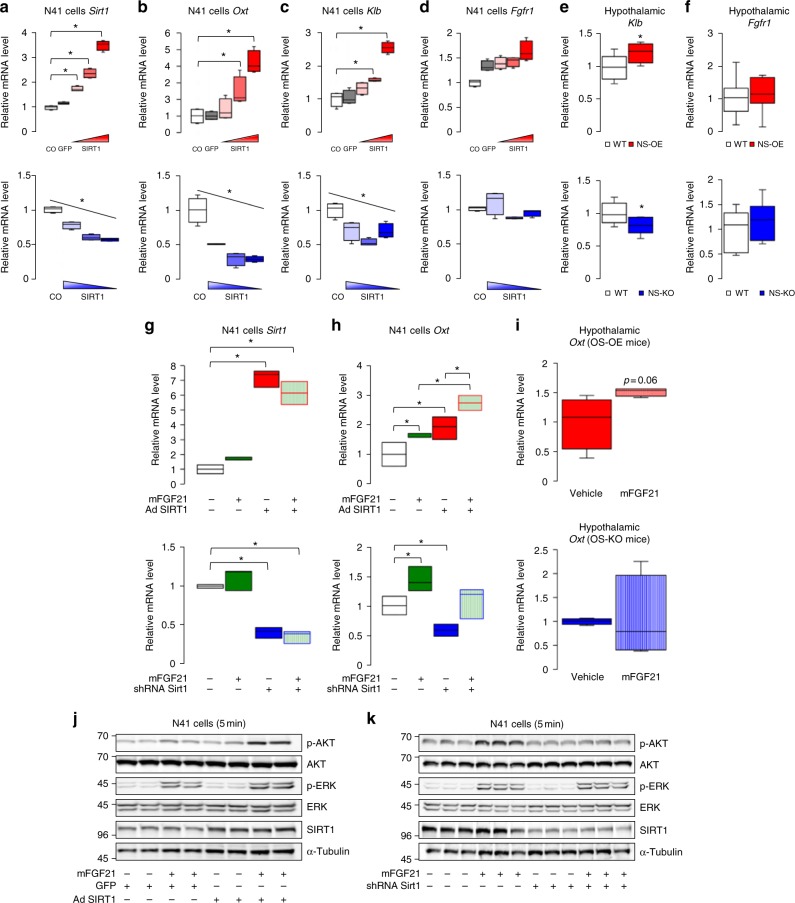
SIRT1 enhances KLB-AKT signalling and promotes FGF21-induced *Oxt* expression. **a**–**d** Effects of Sirt1 manipulation on gene expression in hypothalamic N41 cells. *Sirt1* expression was manipulated by viral vectors to different degrees. Sirt1 expression in untreated (CO), control (GFP) and viral infected cells (red, *Sirt1* overexpressing; blue, *Sirt1* silencing). Shading indicates low (light) to high (dark) MOIs or knockdown efficiencies. Manipulating *Sirt1* expression (**a**) affected expression of *Oxt* (**b**) and *Klb* (**c**), but not *Fgfr1* (**d**) (*n* = 4 per group). **e**, **f** Hypothalamic *Klb* and *Fgfr1* mRNA expression in WT, NS-OE (red) and NS-KO (blue) mice (*n* = 6 per group). **g**, **h** Effects of *Sirt1* overexpression (top) and *Sirt1* silencing (bottom) on FGF21-induced *Oxt* expression. N41 cells were treated with or without 100 nM mFGF21 for 8 h; expression levels of *Sirt1* (**g**) and *Oxt* (**h**) (*n* = 3 per group). **i** Hypothalamic *Oxt* mRNA expression levels in to OS-OE and OS-KO mice after IP injection of mFGF21 (1 mg/kg) or water (vehicle) (*n* = 4 per group). **j**, **k** Effects of overexpressing (**j**) (*n* = 2 per group) or silencing (**k**) (*n* = 3 per group) *Sirt1* expression on FGF21-induced intracellular signalling analysed by western blotting. After viral infection, N41 cells were incubated for 5 min in the absence or presence of 100 nM mFGF21. Data are shown as box and whisker plots (centre line, median; box limits, upper and lower quartiles; whiskers, the minimum and maximum value of a data set). *p < 0.05. Data in **a**–**h** were replicated more than three times, and data in **j**, **k** were replicated twice in the laboratory. See also Supplementary Fig. 6

Our results indicated that SIRT1 regulates simple sugar preference through Oxt and that FGF21 signalling regulates Oxt. Therefore, we hypothesized that SIRT1 may regulate FGF21–Oxt signalling. To analyse the mechanism linking SIRT1 to this FGF21–Oxt signalling, we manipulated SIRT1 levels using viral vectors in the hypothalamic N41 cell line. The level of *Sirt1* expression was positively correlated with levels of *Oxt* and *Klb* expression, but FGF receptor 1 (*Fgfr1*) and *Oxtr* expression was not affected (Fig. 7a–d; Supplementary Fig. 6a). In addition, the expression of *Sirt1* positively correlated with that of *Klb* but not of *Fgfr1* in NS-OE and NS-KO mice hypothalami (Fig. 7e, f), suggesting that SIRT1 promotes *Klb* expression both in vitro and in vivo. Moreover, SIRT1 and FGF21 cooperatively regulated *Oxt* expression in N41 cells (Fig. 7g, h). Additionally, hypothalamic *Oxt* expression was further increased by IP injection of mFGF21 into OS-OE mice but not OS-KO mice (Fig. 7i), suggesting that SIRT1 in Oxt neurons facilitates FGF21-induced *Oxt* expression in vivo. Moreover, SIRT1 overexpression enhanced FGF21-induced AKT phosphorylation, and a *Sirt1* knockdown suppressed it in N41 cells (Fig. 7j, k). Therefore, SIRT1 facilitates FGF21 signalling in Oxt neurons and promotes *Oxt* expression.

Serum FGF21 levels in OS-OE and OS-KO mice were not significantly different from the levels in control mice, either in the ad libitum fed state or 1 h after sucrose ingestion (Supplementary Fig. 6b, c). Therefore, we concluded that simple sugar preference phenotypes in these mice were driven mainly by the altered sensitivity of Oxt neurons to FGF21 and not by changes in the circulating FGF21 levels.

### SIRT1 regulates the expression of Oxt through NRF2

**Fig. 8:**
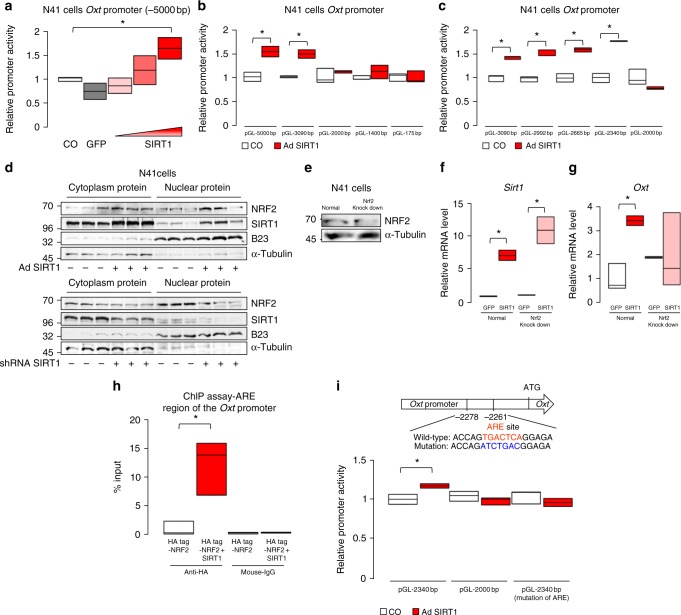
SIRT1 regulates expression of Oxt through NRF2. **a**–**c** Luciferase assays of *Oxt* promoters of various lengths in N41 cells. Luciferase activity was measured in response to adenoviral overexpression of *Sirt1* (ad-Sirt1), the control virus (GFP) and uninfected cells (co, normalized to 1). *Sirt1* activation of full-length (−5000) (**a**) and truncated (**b**, **c**) *Oxt* promoters (*n* = 3 per each group). **d** The effects of overexpressing (upper panels) or silencing (lower panels) *Sirt1* expression in N41 cells on nuclear NRF2 protein levels. Immunoblots of nuclear and cytoplasmic fractions probed for NRF2, SIRT1, B23 (nuclear marker) and α-tubulin (cytoplasm marker) (*n* = 3 per group). **e**–**g** Effect of *Nrf2* knockdown on SIRT1-induced *Oxt* expression. After lentiviral knockdown of *Nrf2* in N41 cells (**e**), *Sirt1* expression levels were manipulated with adenoviral overexpression (**f**), and *Oxt* expression was measured (**g**) (*n* = 4 per group). **h** ChIP performed on HA-tagged NRF2, FLAG-tagged SIRT1 or pCMV5 (empty)-transfected N41 cells using HA tag or mouse IgG antibody. ChIP DNA was subjected to quantitative PCR using primers specific for the ARE region in the *Oxt* promoter. Data are presented relative to input (*n* = 3 per group). **i** Activity of the −2430-bp promoter with and without mutations in the ARE region (*n* = 3 per group). Data are shown as box and whisker plots (centre line, median; box limits, upper and lower quartiles; whiskers, the minimum and maximum value of a data set). *p < 0.05. Data in **a**–**i** were replicated twice in the laboratory. See also Supplementary Fig. 7

To elucidate the mechanisms of transcriptional regulation of the *Oxt* gene, we performed promoter activity assays. Specifically, we aimed to identify the transcription factor(s) responsible for the regulation of *Oxt* expression by SIRT1 and FGF21–KLB–AKT signalling. SIRT1 overexpression enhanced the activity of the −5000 bp *Oxt* promoter (Fig. 8a). DNA deletion studies revealed that a region between −2340 and −2000 bp upstream of the *Oxt* gene is responsible for SIRT1-dependent promoter activation (Fig. 8b, c). Motif analyses showed that this region contains the antioxidant-response element (ARE), which binds the transcription factor NF-E2-related factor 2 (NRF2)^39^. A previous study showed that AKT activation by FGF21 promotes the nuclear translocation and subsequent activation of NRF2^40^. We confirmed that the nuclear NRF2 level was increased with SIRT1 overexpression and decreased with a *Sirt1* knockdown in N41 cells (Fig. 7d).

To address the role of NRF2 in the transcriptional regulation of Oxt by SIRT1 and FGF21, we performed a series of experiments. *Oxt* mRNA expression was significantly upregulated with the NRF2 activator, diethyl maleate (DEM) (Supplementary Fig. 7a, b). A knockdown of NRF2 expression prevented SIRT1-induced *Oxt* expression (Fig. 7e–g). Both SIRT1 overexpression and FGF21 treatment promoted NRF2 recruitment to the single ARE on the *Oxt* promoter (Fig. 8h; Supplementary Fig. 7c), which was detected by chromatin immunoprecipitation (ChIP) assays. Finally, point mutations in the single ARE motif within the −2340 bp *Oxt* promoter construct were sufficient to block SIRT1, FGF21 or DEM-dependent promoter activation (Fig. 8i; Supplementary Fig. 7d, e). Therefore, these data collectively show that SIRT1 promotes Oxt expression through FGF21–KLB–AKT–NRF2 signalling.

### Fasting attenuates FGF21-induced Oxt expression through KLB

**Fig. 9:**
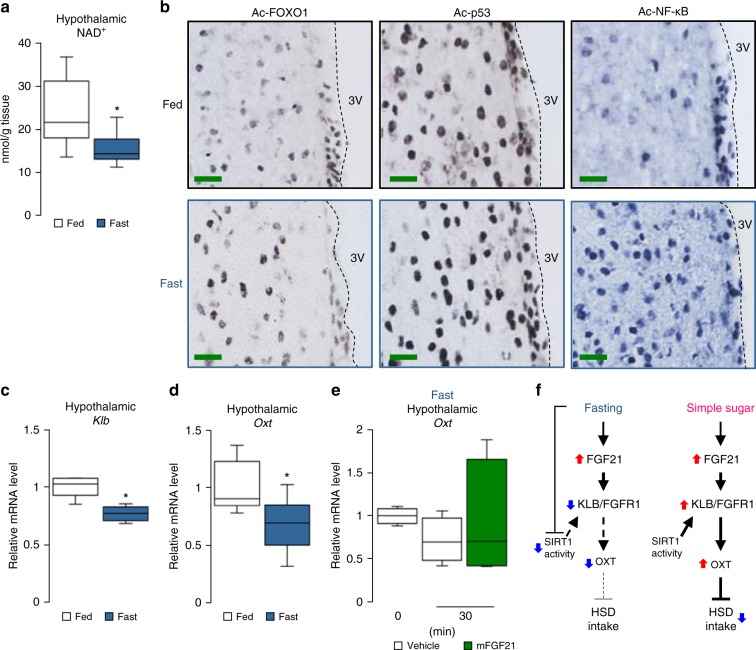
Fasting attenuates hypothalamic SIRT1 activity and hypothalamic FGF21-Oxt signalling. **a** The effects of feeding and fasting on hypothalamic NAD^+^ levels (*n* = 12 in the Fed group, *n* = 13 in the Fasted group). **b** Photomicrographs depict hypothalamus coronal section from wild-type C57BL/6 mice (fed or fasted state) that received 3rd ICV injection of 0.5 μl of TSA (10 μg/μl). The length of scale bars is 100 μm. The sections were immunostained to identify Ac-FOXO1, Ac-p53 and Ac-NF-κB protein (black or blue) expression. **c**, **d** The effects of feeding and fasting on hypothalamic expression levels of *Klb* (**c**) (*n* = 6 in the Fed group, *n* = 7 in the Fasted group) and *Oxt* (**d**) (*n* = 6 in the Fed group, *n* = 7 in the Fasted group) in C57BL6/J mice fed ad libitum or fasted for 24 h. **e** Hypothalamic *Oxt* mRNA expression levels after IP injection of mFGF21 (1 mg/kg) or water (vehicle) in 24-h–fasted C57BL6/J mice (*n* = 4 per group). **f** Schematic diagrams of hypothalamic FGF21–Oxt signalling and SIRT1 in fasting and feeding. (Left) Fasting increased circulating FGF21 levels but suppressed hypothalamic SIRT1 activity, which was associated with the down-regulation of FGF21–KLB–OXT signalling. (Right) During simple sugar feeding, hypothalamic SIRT1 activity was maintained, and FGF21 functioned as a metabolic signal for regulating the simple sugar preference through the FGF21–OXT signalling. Data are shown as box and whisker plots (centre line, median; box limits, upper and lower quartiles; whiskers, the minimum and maximum value of a data set). *p < 0.05. 3V, 3rd ventricle; Ac. acetylated. See also Supplementary Fig. 8

We found the link between FGF21 and Oxt by studying simple sugar preference phenotypes regulated by SIRT1. However, in addition to simple sugar ingestion, plasma FGF21 levels are elevated by fasting^37^, so we analysed the effect of fasting on expression levels of *Klb* and *Oxt* and hypothalamic levels of NAD^+^. Although it is generally assumed that SIRT1 is active during fasting, it has been shown that fasting had differential effects on SIRT1 expression among different tissues^41^. Even within the brain, fasting increased SIRT1 protein levels in the cerebral cortex, but it reduced SIRT1 levels in the hypothalamus^42^. Moreover, we found that fasting also significantly reduced hypothalamic NAD^+^ (Fig. 9a); consequently, as expected, fasting (not caloric restriction) reduced the estimated total hypothalamic SIRT1 activity (estimated by the abundances of SIRT1 protein and its co-factor NAD^+^), leading to increased acetylation levels of SIRT1 substrates (Fig. 9b). It also was associated with reduced expression of *Klb* and *Oxt* (Fig. 9c, d). In contrast to the FGF21-induced *Oxt* expression in fed mice (Fig. 6a), the FGF21-induced *Oxt* expression was not observed in fasted mice (Fig. 9e), indicating that the sensitivity of the hypothalamus to FGF21 is reduced during fasting in association with the reduced expression of FGF21 receptors (due to reduced KLB) (Fig. 9f).

On the other hand, HSD and HFD feeding showed no effect on hypothalamic *Sirt1* mRNA, SIRT1 protein, NAD^+^ levels or the acetylation levels of SIRT1 substrates compared to NC feeding in wild-type C57BL/6 male mice (Supplementary Fig. 8a–d). Consequently, under the fed condition (when hypothalamic SIRT1 activity was intact), the FGF21–Oxt signalling pathway was intact, and it negatively regulated the simple sugar preference through Oxt (Fig. 9e). SIRT1 may serve as a tuner for FGF21 sensitivity in Oxt neurons and regulate simple sugar preference in a context-dependent manner.

## Discussion

**Fig. 10:**
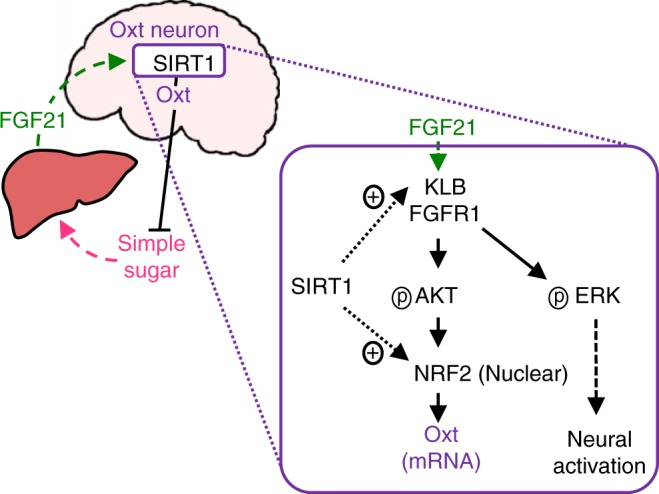
Proposed model of simple sugar preference regulation by neuronal SIRT1. Ingestion of simple sugar promotes secretion of FGF21 from the liver. The hepatokine FGF21 acts as an endocrine signal to Oxt neurons and promotes *Oxt* transcription (AKT signalling) and neuronal activation (ERK signalling). Oxt suppresses simple sugar preference by acting on OXTR. SIRT1 promotes FGF21 sensitivity in Oxt neurons and stimulates *Oxt* transcription through NRF2, thereby potentiating the negative feedback regulation of simple sugar preference through FGF21 and Oxt

We identified SIRT1 as a regulator of macronutrient preference and found that it regulates simple sugar preference through FGF21–NRF2–Oxt signalling (Fig. 10). Oxt neurons were identified as one of the in vivo targets of FGF21, and FGF21 promoted Oxt neuronal activation via ERK signalling and Oxt expression via AKT signalling. SIRT1 promoted FGF21 sensitivity in Oxt neurons and enhanced negative feedback regulation of simple sugar preference. Thus, our findings extend understanding of the homoeostatic regulation of macronutrient preference. Considering the importance of macronutrient balance on health span and weight control, greater understanding of this aspect of feeding regulation would help with elucidating the physiology and pathophysiology of feeding behaviour in relation to health and diseases.

Obesity is accompanied by FGF21 resistance, both in the periphery and in the brain, due to receptor downregulation and reduced FGF21 transfer to cerebrospinal fluid^43–45^. Central FGF21 resistance may be among the overlooked underlying pathologies contributing to aberrant dietary choices that exacerbate obesity and diabetes. Of interest, hepatic SIRT1 promotes FGF21 transcription^46^. Thus, systemic activation of SIRT1 may promote systemic FGF21 signalling by increasing the endocrine supply of the ligand (FGF21) from the liver and increasing expression of the co-receptor (β-klotho) in the target organs. These changes would lead to enhanced intracellular signalling in FGF21 target cells, such as in Oxt neurons, as shown in this work. Obese people and patients with diabetes have decreased circulating Oxt levels^47^, and intranasal Oxt treatment causes a shift in metabolism from using carbohydrates to using fat^48^. Based on our finding that central FGF21 signalling stimulates Oxt neurons and suppresses simple sugar preference, we speculate that improving central FGF21 sensitivity could improve diet selection to match the peripheral tissue substrate need through Oxt and improve health in obese and diabetic people. Furthermore, this improvement could elevate stress resilience by promoting Oxt action because Oxt ameliorates the stress response^49^.

We also found that multiple longevity genes co-ordinately regulated the simple sugar preference in mice. Both FGF21 and NRF2 are longevity genes that extend the lifespan in model organisms^50–53^. SIRT1 may promote a long lifespan in part by coordinating multiple longevity pathways, including previously described targets FOXO^54^ and HSF1^55^. Furthermore, Oxt attenuates stress responses^49^. Metabolic shifts, altered diet selection and improved stress resilience may represent an integrated biological strategy for survival against physical and psychological stressors. Other evolutionarily conserved energy sensor molecules that can cause a metabolic switch between glycolysis and fatty acid oxidation in peripheral tissues may work in the nervous system in a similar manner.

How some neuropeptides regulate feeding behaviour in a macronutrient-specific manner remains elusive. One potential mechanism is that release of such neuropeptides may be triggered only by the macronutrient-specific signals, such as FGF21 regulating Oxt, as shown here. Another potential mechanism is that neuropeptides may regulate particular projection target(s) in a macronutrient-specific manner. For instance, Oxt neurons project to multiple sites within the reward system, including the NAc, amygdala and VTA^22^. Oxt projections to NAc^56^ and VTA^57^ have been implicated in the regulation of sucrose preference, and the VTA projection gates social reward^58^. Oxt neurons also project to other brain areas; intranasal administration of Oxt in humans suppresses hypothalamic activation to visual food cues^59^, and enhanced cognitive control in a food craving task accompanied by neural activation of the prefrontal cortex, which is involved in inhibitory control^60^. Because only a fraction of PVH Oxt neurons responded to FGF21 in the current work, analysing the projection sites of FGF21-responsive PVH Oxt neurons may improve understanding of how Oxt controls simple sugar preference at the neurocircuit level. Indeed, Oxt neural projections might selectively reduce the hedonic value of sugars.

Here we identified the link between FGF21 and Oxt by focusing on the metabolic signals that are trigged by simple sugar ingestion. We aimed to address the role of taste versus metabolic signals (post-ingestive effect) by using non-nutritive artificial sweeteners. When we performed the 2-bottle tests in sugar-naive mice, we did not observe any differences in the preferences for the sweeteners in both OS-OE and OS-KO mice (Fig. 4f, g). However, when the same 2-bottle tests were performed with mice that had experience sugar previously, we found that the preference for saccharin was similarly affected in both OS-OE and OS-KO mice (Supplementary fig. 9). It has been known that non-nutritive artificial sweeteners could have post-ingestive metabolic effects called cephalic phase responses^61^. It is a form of conditioning-based learning, in which the taste cue (sweet taste) experience upon sugar ingestion become associated with the post-ingestive metabolic experience, so that anticipatory pre-absorptive metabolic responses (such as salivation, gastric acid secretion, secretion of metabolic hormones, and thermogenesis) are induced by the conditioned taste cue^30,31^. Therefore, performing two-bottle experiments in sugar-naive mice was important for addressing the role of taste versus metabolic signals in our cases. Although both FGF21 and Oxt had been implicated in the preferences to artificial sweeteners without affecting taste reactivity^24,32,34,62,63^, it is not clear if these past works were performed in sugar-naive animals. Pharmacological administration of recombinant FGF21 or FGF21 analogue decreased preferences to sugars and sweeteners^32,34^. However, *Fgf21* Tg mice showed decreased preferences to both sugar and saccharin^34^, while *Fgf21* KO mice showed increased preference to sugars but not saccharin^32^. Another possible explanation for the differences in the sweetener preferences between our sugar-naive mice and the past literature is microbiome. The artificial sweeteners altered microbiome and disturbed systemic glucose homoeostasis^64,65^, and *Lactobacillus reuteri* increased PVH oxytocin in mice^66^, suggesting that the preference for the artificial sweeteners could be regulated through microbiome and Oxt. The differences in microbiome between our sugar-naive mice and mice used in the past literature may have caused the differences in the preferences to sweeteners. Therefore, the mechanisms that regulate preferences to sweeteners remain elusive.

This study also did not resolve the molecular mechanisms underlying the regulation of fat preference by SIRT1. At the least, Oxt is not responsible for the regulation of fat preference in NS-OE and NE-KO mice because SIRT1 manipulation in Oxt neurons (OS-OE and OS-KO mice) did not affect fat preference (Fig. 3f). A potential mechanism might operate by improving the sensitivity to metabolic signals because fat intake drives a positive feedback loop^21^ and SIRT1 improves central sensitivity to hormones such as FGF21 and leptin^10,11^. Furthermore, different fatty acids have different effects on health; thus, SIRT1 may modulate preferences for particular types of fatty acids. Indeed, we found that SIRT1 modulated only the preference for simple sugars, not all carbohydrates. Our understanding of homoeostatic diet selection therefore might be advanced by studies that focus on identifying fatty acid preferences under SIRT1 regulation.

In summary, our investigation of the mechanisms that regulate macronutrient-based diet selection unexpectedly revealed that Oxt is regulated by SIRT1, FGF21 and NRF2. Understanding the homoeostatic mechanisms for macronutrient selection may unveil an underlying pathophysiology of aberrant diet selection in obesity and lead to the development of interventions that promote healthy diet choice.

## Methods

### Animals

Mice were generated to overexpress neuron-specific *Sirt1* (NS-OE) and Oxt-neuron-specific *Sirt1* (OS-OE). These strains were generated by breeding mice that harboured *Rosa26*-*Sirt1*^10^ with mice that harboured neuron-specific *Tau*-*Cre*^15^(for NS-OE) or Oxt-neuron-specific *Oxt-ires-Cre*^28^(for OS-OE). Mice were also generated to knock out neuron-specific *Sirt1* (NS-KO) and Oxt-neuron-specific *Sirt1* (OS-KO). These strains were generated by breeding mice that harboured a floxed *Sirt1* allele^16^ with mice harbouring either *Tau*-*Cre* recombinase (NS-KO) or *Oxt-ires-Cre* (OS-KO) recombinase. *Rosa26*-*Sirt1* mice had a 129/Bl6 mixed background, maintained with three backcrosses to Bl6. *Oxt-ires-Cre* transgenic mice had a 129/Bl6 mixed background, maintained with five backcrosses to Bl6. *Tau*-*Cre* and *Sirt1-flox* mice had a pure C57BL6/J genetic background.

All animals were maintained in a specific pathogen-free space under a 12-h light/dark regimen. At the time of the experiments, mice were 10–16 weeks old. Unless otherwise specified, male littermates were randomly assigned to all experimental groups. The NC (CE-2; CREA Japan, Tokyo, Japan), HSD (F2HScD; Oriental Yeast Co. Ltd, Tokyo, Japan), HFD (HFD32; Oriental Yeast Co. Ltd, Tokyo, Japan), and water were provided ad libitum. Intakes of NC, HSD and HFD and body weight were measured daily after 11 weeks of age. None of the animals included in the data analyses displayed any health impairments.

All experimental procedures were performed in accordance with the Guide for the Care and Use of Laboratory Animals of the Science Council of Japan and approved by the Animal Experiment Committee of Gunma University.

### Two-food choice studies

For the two-food choice studies, 10-week-old male or female mice with wild-type, NS-OE, NS-KO, OS-OE or OS-KO genotypes were acclimated for 1 week to feeding from multifeeders (Shinfactory, Fukuoka, Japan). Then, mice were given access to a multifeeder that provided either NC vs. HSD or NC vs. HFD. Food intake and body weight were measured daily for 5 days (*n* = 6 per group). The positions of the two food choices were switched every day to exclude positional effects.

### Long and brief two-bottle choice studies

For the two-bottle choice studies, 10-week-old male mice with wild-type, NS-OE, NS-KO, OS-OE or OS-KO genotypes were first acclimated to cages with two bottles (Drink-O-Meter) that contained water for 1 week. For the long two-bottle choice studies, mice were given ad libitum access to one bottle with water and one bottle with a test solution for 3 days (*n* = 7 in the WT group; *n* = 6 in the OS-OE and OS-KO groups). The test solution contained one of the following: 100 mM glucose (Wako, Osaka, Japan), 100 mM fructose (Wako, Osaka, Japan), 100 mM sucrose (Nakarai, Kyoto, Japan), 2% dextrin (Nakarai, Kyoto, Japan), 0.2% saccharin (Wako, Osaka, Japan), 0.2% aspartame (Tokyo Chemical Industry, Tokyo, Japan) or 1.5 mM quinine (Wako, Osaka, Japan). NC was also available *ad libitum* throughout the test period. The positions of the two bottles were changed every day to exclude positional effects.

For the brief two-bottle choice studies, mice were given ad libitum access to one bottle with water and one bottle with 100 mM sucrose or 0.2% saccharin solution for only 10 min (*n* = 6 per group). NC was also available ad libitum throughout the test period. The positions of the two bottles were changed every 3 min to exclude positional effects.

### Effect of Oxtr antagonist on sucrose selection behaviour

Eleven-week-old male mice with wild-type, NS-OE and OS-OE genotypes were injected IP with the Oxtr antagonist OXTR-A (L-368, 899, 5 mg/kg, twice a day for 3 days; Tocris Bioscience, Bristol, UK) or water (vehicle). After the OXTR-A treatment was initiated, mice were presented with two food choices (NC vs. HSD) in multifeeders. Food intake was measured daily for 3 days (*n* = 5 per group for wild-type vs. NS-OE; *n* = 6 per group for wild-type vs. OS-OE). The positions of the two foods in the multifeeders were changed every day to exclude positional effects.

### Indirect calorimetry and locomotor activity measurement

Oxygen consumption and CO_2_ production were measured in individual male OS-KO mice (control, *n* = 6; KO, *n* = 6) and OS-OE mice (control, *n* = 6; OE, *n* = 7) at 8 weeks of age, with an Oxymax apparatus (Columbus Instruments, Columbus, OH, USA). The O_2_ and CO_2_ measurements were performed every 18 min for each mouse over a 3-day period and the data from the final day were analysed. Locomotor activity was measured with the ACTIMO-100 (Shinfactory).

### Generation of adeno-associated viral (AAV) vector

AAV Helper-Free System (Agilent Technologies, Inc., Santa Clara, CA, USA) were used for the generation of the AAV2-hSyn-FLEX-mCherry or AAV2-hSyn-FLEX-hM4Di-mCherry viral vector. Purification method was modified from the previously published protocol^67^. Briefly, HEK293 cells were transfected with a pAAV-hSyn-FLEX-mCherry or pAAV-hSyn-FLEX-hM4Di-mCherry vector plasmid (gifts from Dr. Bryan Roth at the University of North Carolina, USA), pHelper and pAAV-RC2 (purchased from Cell Biolabs Inc, San Diego, CA, USA) using a standard calcium phosphate method. Three days after transfection, cells were collected and suspended in artificial CSF solution containing (in mM) 124 NaCl, 3 KCl, 26 NaHCO_3_, 2 CaCl_2_, 1 MgSO_4_, 1.25 KH_2_PO_4_ and 10 d-Glucose. Following four freeze–thaw cycles, the cell lysates were treated with Benzonase nuclease (Merck, Darmstadt, Germany) at 45 °C for 15 min, then centrifuged twice at 20,000×*g* for 10 min at 4 °C. The supernatant was used as the virus-containing solution. Quantitative PCR was performed to measure the titre of purified virus. Virus aliquots were then stored at −80 °C until use for experiment.

### DREADD experiment

AAV2-hSyn-FLEX-mCherry or AAV2-hSyn-FLEX-hM4Di-mCherry virus was injected into and specifically expressed in PVH Oxt neurons. The skulls of 10-week-old male OS-OE mice were exposed and a small hole drilled above each side of the PVH. Bilateral injection was performed with a Hamilton 10-μl syringe (Sigma Aldrich, St Louis, MO, USA) filled with 1 μl of virus (9.94 × 10^11^ copies/ml) at the following coordinates: bregma: AP:−0.82 mm; DV:−4.75 mm; and RL: ± 0.25 mm. The injection speed was controlled at 100 nl/min with a micromanipulator (IMS20, Narishige, Tokyo, Japan). Experiments were performed 2 weeks post-injection to allow for recovery and viral expression.

The AAV2-hSyn-FLEX-mCherry virus—or AAV2-hSyn-FLEX-hM4Di-mCherry-expressing mice were injected IP with clozapine (1 μg/kg) or PBS (pH 7.4, vehicle) once a day at 13:00, and mice then were given access to a multifeeder that provided either NC vs. HSD. Food intake was measured daily for 3 days (*n* = 8 per group). The positions of the two food choices were switched every day to exclude positional effects. We used the injection of clozapine instead of clozapine-N-oxide (CNO), because upon systemic CNO injections, converted clozapine readily enters the brain and occupies central nervous system-expressed DREADD receptors; in contrast, systemic subthreshold clozapine injections induce preferential DREADD-receptor-mediated behaviours^29^.

After the completion of study period, mice were anesthetized, and then perfused transcardially, first with PBS (pH 7.4), then with 4% paraformaldehyde (PFA). Dissected brains were post-fixed in a 4% PFA solution at 4 °C overnight, then incubated in a 20% sucrose solution at 4 °C overnight. Brains were sectioned coronally, at a thickness of 25 μm. Sections were collected in PBS, pH 7.4, transferred to a cryoprotectant solution, and stored at −20 °C.

For immunostaining, sections were incubated with 3% normal donkey serum (NDS) in PBS containing 0.3% Triton X-100 (3% NDS/0.3% PBT) for 1 h. Then we added rabbit anti-Oxt antibody (1:800; #T-4087.0050, Peninsula Laboratories, San Carlos, CA, USA) and rat anti-mCherry antibody (1:600; #16D7, Thermo Fisher, USA) diluted in 3% NDS/PBT, and sections were incubated overnight at 4 °C. After washing in PBS that contained 0.3% Triton X-100, sections were incubated for 1 h with Alexa Fluor 488 Donkey anti-rabbit IgG (1:400; R37118, Thermo Fisher Scientific, Waltham, MA, USA) and rat anti-Cy3 antibodies (1:200; Jackson ImmunoReseach, West Grove, PA, USA) diluted in 3% NDS/0.3% PBT. Fluorescence images were acquired with a BZ-9000 fluorescence microscope (KEYENCE, Osaka, Japan).

### Double staining immunohistochemistry

For c-Fos/Oxt double-staining, 11-week-old male C57BL/6 mice were injected IP with murine FGF21 (mFGF21; 1 mg/kg; BioVendor, Modrice, Czech Republic) or water (vehicle) (*n* = 4 per group). At 2 h after the injection, mice were anaesthetized with sodium pentobarbital (50 mg/kg, IP) and perfused transcardially with ice-cold 0.05 M PBS (pH 7.4) followed by a fixation solution containing 4% PFA. Dissected brains were post-fixed in 4% PFA solution at 4 °C overnight, then incubated in 20% sucrose solution at 4 °C overnight. Brains were sectioned coronally, at a thickness of 25 μm, and stored in cryoprotectant solution at −30 °C until use. We performed double immunostaining of c-Fos and Oxt as follows. Sections were washed in PBS, then treated with 0.3% H_2_O_2_ diluted in PBS for 15 min. Sections were blocked with 3% NDS diluted in PBS containing 0.25% Triton X-100 for 30 min. The sections were incubated in rabbit anti-c-Fos antiserum (1:25,000; #ABE457, Merck Millipore, Billerica, USA) diluted in blocking solution overnight at 22–25 °C. After being washed in PBS, the sections were incubated with biotinylated goat anti-rabbit IgG (1:400; BA-1000, Vector Laboratories, Burlingame, CA, USA) for 40 min and incubated with ABC reagent (Vector Laboratories) for 40 min. The sections were washed in PBS and 0.175 M sodium acetate buffer (pH 5.6), and colour was developed with a nickel-diaminobenzidine solution (10 g/l nickel ammonium sulphate, 0.2 g/l diaminobenzidine and 0.006% H_2_O_2_ in sodium acetate buffer). After being washed in PBS, the sections were treated with 0.3% H_2_O_2_ diluted in PBS for 15 min and blocked with blocking solution. Incubation followed in rabbit anti-Oxt antibody (1:1200) diluted in blocking solution overnight at 22–25 °C. After another wash in PBS, the sections were incubated with biotinylated goat anti-rabbit IgG (1:400) for 40 min and incubated with ABC reagent (Vector Laboratories) for 40 min. After a final wash in PBS and 0.1 mM Tris-HCl buffer (pH 7.5) containing 0.15 mM, colour was developed with a diaminobenzidine solution.

For Oxt/SIRT1 double staining of WT and OS-OE mouse brain sections, the sections were incubated with 3% NDS in PBS containing 0.1% Triton X-100 (3% NDS/0.1% PBT) for 1 h and then overnight at 4 °C with rabbit anti-OXT antibody (1:800) and mouse anti-SIRT1 antibody (1:200; #ab110304, Abcam, Cambridge, UK) diluted in 3% NDS/0.1% PBT. After washing in 0.1% PBT, sections were incubated for 1 h with Alexa Fluor 488 Donkey anti-Rabbit IgG (1:400) and Alexa Fluor 594 Donkey Anti-Mouse IgG (1:400; R37115, Thermo Fisher Scientific) diluted in 3% NDS/0.1% PBT. Fluorescence images were acquired using BZ-9000 (KEYENCE, Osaka, Japan).

For Oxt/SIRT1 double staining of WT and OS-KO mouse brain sections, the sections were washed in PBS, and then treated with 3% H_2_O_2_ diluted in PBS for 5 min. Sections were blocked with 3% NDS/0.1% PBT for 30 min. The sections were incubated in mouse anti-SIRT1 antibody (1:1000) diluted in blocking solution overnight at 22–25 °C. After washing in PBS, the sections were incubated with labelled polymer-HRP (K4061, Agilent Technologies, California, USA) for 30 min. The sections were washed in PBS and color was developed with a nickel-diaminobenzidine solution (10 g/l nickel ammonium sulphate, DAB substrate kits (K3468, Agilent Technologies, California, USA)). After washing in PBS, the sections were treated with 3% H_2_O_2_ diluted in PBS for 5 min. The sections were blocked with blocking solution. The section were incubated in rabbit anti-OXT (1:800) diluted in blocking solution overnight at 22–25 °C. After washing in PBS, the sections were incubated with labelled polymer-HRP for 30 min. The sections were washing in PBS and color was developed with DAB substrate kits.

### Evaluation of the acetylation levels of SIRT1 substrates

In order to detect the difference in acetylation status of SIRT1 substrates in mice, they were anesthetized and 0.5 μl of trichostatin A (10 μg/μl) was injected into the third ventricle at the following coordinates: bregma: AP:−1.70 mm; DV:−5.3 mm; and RL:0.00 mm. At 1 h after the injection, mice were anesthetized with sodium pentobarbital (50 mg/kg, IP) and perfused transcardially with ice-cold 0.05 M PBS (pH 7.4) followed by a fixation solution containing 4% paraformaldehyde (PFA). Dissected brains were post-fixed in 4% PFA solution at 4 °C overnight, then they were incubated in 20% sucrose solution at 4 °C overnight. Brains were sectioned coronally, at a thickness of 25 μm, and stored in cryoprotectant solution at −30 °C until use.

For Ac-FOXO1, Ac-p53 and Ac-NF-κB immunostaining of WT, NS-OE and C57BL/6 (fed NC, HSD or HFD; and fed or fasted state) mouse brain sections, the sections were washed in PBS, and then treated with 3% H_2_O_2_ diluted in PBS for 5 min. Sections were blocked with 3% NDS/0.1% PBT for 30 min. The sections were incubated in rabbit anti-Ac-FOXO1 antibody (1:250; #sc-49437-R, Santa Cruz Biotechnology), rabbit anti-Ac-p53 antibody (1:150; #2525, Cell Signaling) and rabbit anti-Ac-NF-κB antibody (1:400; #19870, Abcam) diluted in blocking solution overnight at 22–25 °C. After washing in PBS, the sections were incubated with labelled polymer-HRP (K4061, Agilent Technologies) for 30 min. The sections were washed in PBS and color was developed with a nickel-diaminobenzidine solution.

For Oxt/Ac-FOXO1, Ac-p53 and Ac-NF-κB double staining of WT and OS-OE mouse brain sections, the sections underwent the same procedures as described above. After the development of the acetyl substrate signals, the sections underwent the following additional steps for Oxt staining. After washing in PBS, the sections were treated with 3% H_2_O_2_ diluted in PBS for 5 min. The sections were blocked with blocking solution. The section were incubated in rabbit anti-OXT (1:1,000; #T-4087.0050, Peninsula Laboratories, San Carlos, CA, USA) diluted in blocking solution overnight at 22–25 °C. After washing in PBS, the sections were incubated with labelled polymer-HRP for 30 min. The sections were washing in PBS and color was developed with DAB substrate kits.

### Combined in situ hybridization and immunohistochemistry

Brain sections were prepared from 11-week-old male C57BL/6 mice, as described above for the double-staining immunohistochemistry. In situ hybridization of *β-Klotho* (*Klb*) was performed as follows. Sections were washed in 0.1 M phosphate buffer (pH 7.0), then treated with acetylation buffer (pH 8.0) for 10 min. Next, the sections were hybridized with 0.5 mg/ml digoxigenin-labelled sense and antisense RNA probes specific for the *Klb* sequence (probe sequences are listed in Supplementary Table 2). The sections and antibodies were incubated in diluted hybridization buffer (50% [v/v] formamide, 20 × SSC, 10% blocking reagent, 2% N-lauroylsarcosine [NLS] and 10% sodium dodecyl sulphate) for 16–24 h at 60 °C. Hybridized sections were washed twice at 60 °C for 20 min with a mixture of 2 × SSC, 50% (v/v) formamide, and 0.1% NLS. Then, sections were washed twice at 37 °C for 20 min with a mixture of 2 × SSC and 0.1% NLS, followed by two more washes at 37 °C for 20 min with a mixture of 0.2 × SSC and 0.1% NLS. Subsequently, the sections were incubated for 5 h at 22–25 °C with alkaline phosphatase-conjugated anti-digoxigenin antibody (1093274, Roche, Indianapolis, IN, USA), which was diluted at 1:1000 with 1% blocking reagent. Incubated sections were washed 3 times for 15 min with a mixture of 0.1 M Tris-HCl (pH 7.5), 0.15 M NaCl and 0.05% Tween 20. Then, sections were incubated for 16–24 h at 37 °C with NBT/BCIP stock solution (Roche), diluted at 1:50 with a mixture of 0.1 M Tris-HCl (pH 7.5), 0.15 M NaCl and 0.05% Tween 20. After two washes with a mixture of PBS and 1 mM EDTA for 5 min, the sections were washed for 24 h at 37 °C with a mixture of 0.1 M Tris-HCl (pH 7.5), 0.15 M NaCl and 0.05% Tween 20. A PBS wash followed, then treatment with 0.3% H_2_O_2_ diluted in PBS for 15 min. The sections were then blocked with 1% bovine serum albumin and 2% normal goat serum diluted in PBS with 0.25% TritonX-100 for 30 min. The sections were incubated overnight with rabbit anti-Oxt antibodies (1:1200) diluted in blocking solution. After being washed in PBS, the sections were incubated with biotinylated goat anti-rabbit IgG (1:400) for 40 min, then incubated with ABC reagent for 40 min. The sections were washed three times in PBS, and colour was developed with a diaminobenzidine solution (0.2 g/l diaminobenzidine and 0.006% H_2_O_2_ in PBS).

### Single-neuron cytosolic Ca^2+^ concentration measurement

Single neurons were prepared according to procedures reported previously^68^ with slight modifications. Briefly, single neurons were prepared from 5-week-old male C57BL/6 mice. The dissected tissues were washed with 10 mM HEPES-buffered Krebs-Ringer bicarbonate buffer (HKRB) containing 10 mM glucose. They were then incubated in HKRB supplemented with 20 U/ml papain (P4762, Sigma Aldrich), 0.015 mg/ml DNase II (D-4138, Sigma Aldrich) and 0.75 mg/ml bovine serum albumin (A2153, Sigma Aldrich) for 16 min at 36 °C. This incubation was followed by gentle mechanical trituration. Then, the cell suspension was centrifuged at 100 × *g* for 5 min. The pellet was resuspended in HKRB and distributed onto coverslips.

Cytosolic Ca^2+^ concentration ([Ca^2+^]_i_) was measured by ratiometric fura-2 fluorescence imaging. Briefly, after incubation with 2 μM fura-2/AM (F016, Dojindo, Kumamoto, Japan) for 40 min, the cells were mounted in a chamber and superfused with HKRB at 1 ml/min at 33 °C. Moreover, mFGF21 (50 ng/ml) was administered in the superfusion solution, and [Ca^2+^]_i_ was measured for 5 min after addition of mFGF21. Fluorescence images from excitation at 340 and 380 nm were detected every 10 s with a cooled charge-coupled device camera (ORCA-R2 C10600, Hamamatsu Photonics, Shizuoka, Japan), and the ratio image was produced by an Aquacosmos (Hamamatsu Photonics).

The single neurons subjected to [Ca^2+^]_i_ measurements were subsequently immunostained for Oxt using anti-Oxt antibodies. Briefly, the neurons were post-fixed in 4% PFA solution at 4 °C overnight. Rabbit anti-Oxt antibodies (1:1000) were used as primary antibodies, and Alexa Fluor 594 Donkey Anti-Rabbit IgG (1:400; A-11055, Thermo Fisher Scientific) was used as secondary antibody, respectively. Fluorescence images were acquired using an Eclipse TE2000-U (Nikon, Tokyo, Japan).

### Effects of mFGF21 on hypothalamic gene expression

Eleven-week-old male C57BL/6 mice were injected IP with mFGF21 (1 mg/kg) or water (vehicle) (*n* = 4 per group). At 30 min after the injection, mice were killed and the hypothalamus was harvested for gene expression analyses.

### Cell culture

Mouse embryonic hypothalamic N41 cells (Cosmo Bio, Tokyo, Japan) were maintained in high-glucose DMEM (Thermo Fisher Scientific) supplemented with 10% foetal bovine serum (Thermo Fisher Scientific).

### Adenoviral and lentiviral infections

N41 cells were plated for 24 h before infections. In the *Sirt1* overexpression experiments, N41 cells were infected with an adenovirus that carried a wild-type *Sirt1* or *GFP* (green fluorescent protein; vector control). Infections were performed at a multiplicity of infection (MOI) of 100 to 600 infectious units per cell, and the infections were carried out in culture medium for 24 h at 37 °C. In the *Sirt1* and *Nrf2* knockdown experiments, N41 cells were infected with fresh lentivirus that carried one of two *Sirt1* short hairpin RNAs (TRCN0000306512 or TRCN0000326966, Sigma Aldrich) or the *Nrf2* short hairpin RNA (TRCN0000012132, Sigma Aldrich), respectively. Infections were carried out in DMEM containing 8 μg/ml polybrene for 24 h. Then, cells were cultured for an additional 72 h. Infected cells were selected based on puromycin resistance (0.5 μg/ml; 7 days). After selection, virus-infected cells were seeded in 24-well plates with culture medium at a density of 1 × 10^5^ cells/well, incubated for 24 h, then subjected to serum starvation for 24 h before testing the effect of mFGF21 treatment to avoid the influence of various hormones contained in serum.

### mFGF21, DEM and MAPK inhibitor treatments

N41 cells were seeded in 24-well plates with culture medium at a density of 1 × 10^5^ cells/well and incubated for 24 h, followed by serum starvation for 24 h. Then, cells were untreated or treated with either 100 nM mFGF21 or 100 μM DEM (NRF2 activator; Wako) for 0, 2, 4 or 8 h. In an additional experiment, the cells were treated for 8 h with different concentrations of mFGF21 (0, 25, 50 or 100 nM) or DEM (0, 25, 50 or 100 μM) (*n* = 3 per group).

In kinase inhibitor experiments, N41 cells were untreated or treated with either ERK inhibitor (10 μM U0126; Sigma Aldrich) or PI3K inhibitor (10 μM wortmannin; Sigma Aldrich) for 30 min. Then, cells were further incubated for 8 h with or without addition of 100 nM mFGF21 (*n* = 4 per group).

### Quantitative PCR analysis

Total RNA was isolated from the dissected hypothalamus or from N41 cells with RNAiso Plus (Takara Bio, Kyoto, Japan). The isolated RNA was reverse transcribed to cDNA with the Improm II Reverse Transcription System (Promega, Fitchburg, WI, USA). The generated cDNA samples (1 μg) were subjected to real-time PCR with the Light Cycler system and Light Cycler 480 SYBR Green I (Roche). The target mRNA levels were expressed relative to the levels of mouse *β-actin*. The primer sequences are listed in Supplementary Table 1.

### Immunoblot analysis

Total cell lysates and nuclear lysates were prepared using lysis buffer supplemented with a Complete Mini Protease Inhibitor Cocktail tablet (Roche), 100 μM MG132 (Sigma) and 0.25 mg/ml ubiquitin aldehyde (Peptide Institute, Osaka, Japan). To prepare cytoplasmic and nuclear lysates, ice-cold cytoplasmic lysis buffer (5 mM PIPES (KOH) pH 8.0, 65 mM KCl and 0.5% NP 40) was added and the supernatants after centrifugation recovered as cytoplasmic lysates. Remaining nuclear pellets were mixed with the lysis buffer to make nuclear lysates. The lysates were resolved with polyacrylamide gel electrophoresis, then the separated proteins on the gel were transferred to nitrocellulose membranes. The membranes were probed with primary antibodies against SIRT1 (1:1000; #07–131, Millipore), ERK1/2 (1:1000; #9102, Cell Signaling Technology, Danvers, MA, USA), phospho-ERK1/2 (1:1000; #4376, Cell Signaling Technology), AKT (1:1000; #9272, Cell Signaling Technology), phospho-AKT (1:1000; #9271, Cell Signaling Technology), NRF2 (1:1000; #ab31163, Abcam, Cambridge, UK), α-tubulin (1:500; #sc-5286, Santa Cruz Biotechnology, Dallas, TX, USA) and B23 (1:200; #sc-6013, Santa Cruz Biotechnology). Then, membranes were incubated with corresponding horseradish peroxidase–conjugated secondary antibodies. Immunoreactive proteins were assessed with a LAS-4000 Image analyser (Fuji Film, Tokyo, Japan) and densitometry with ImageJ software (US National Institutes of Health).

### Luciferase assays

For luciferase reporter assays, putative promoter regions of the *Oxt* gene were amplified from genomic DNA and cloned upstream of the firefly luciferase gene in the pGL3 plasmid (Promega). We cloned different-sized fragments of DNA upstream of the wild-type *Oxt* open reading frame (-5000, -3090, -2992, -2665, -2340, -2000, -1400 and -175 bp) into a pGL3 basic vector. To pinpoint the promoter, we mutated all the base pairs in the antioxidant-response element (ARE; 5′-TGACTCA-3′ → ATCTGAC) site, at position −2273 to −2266. This fragment was also cloned into the pGL3 vector. We performed transfections of the pGL3-*Oxt* promoter or the pGL3-mutated *Oxt* promoter into cultured N41 cells with Amaxa nucleofection (Lonza, Basel, Switzerland), according to the manufacturer’s instructions. The cells (1 × 10^5^ cells/well) were seeded in 24-well plates before transfections. Firefly luciferase and *Renilla reniformis* luciferase activities were measured with the Dual Luciferase Reporter assay system (Promega), according to the manufacturer’s instructions. Firefly luciferase activities were always normalized to *Renilla reniformis* activities (*n* = 3 per group).

### Chromatin immunoprecipitation assay

N41 cells were transfected using the Lipofectamine 3000 reagent (Thermo Fisher Scientific) according to the manufacturer’s instructions. Where indicated, expression plasmids encoding HA-tagged NRF2, FLAG-tagged SIRT1 or pCMV5 (empty) were cotransfected. Chromatin was extracted from N41 cells transfected with plasmids using the Chromatin Extraction Kit (ab117152; Abcam) (*n* = 3 per group). The lysates then were sonicated to shear the chromatin to 500 and 1000 bp, keeping the samples ice cold. Immunoprecipitation was performed overnight at 4 °C with specific antibodies. After immunoprecipitation, DNA was heated at 65 °C overnight to reverse formaldehyde cross-linking. DNA fragments then were purified with a QIAquick Gel Extraction Kit (Qiagen, Hilden, Germany). Specific antibodies against the HA tag (3F10; Roche) were used for the immunoprecipitation. Normal mouse IgG antibody (sc-2025; Santa Cruz Biotechnology) served as a negative control. Precipitated DNA fragments were analysed by PCR amplification using primers directed against the ARE region in the Oxt promoter.

### FGF21 level measurements

Eleven-week old, male WT, OE-OS and OS-KO mice fed NC ad libitum (*n* = 5 per group) were killed, blood was collected and serum was prepared. Eleven-week-old, male OS-KO mice (control, *n* = 6; KO, *n* = 6) and OS-OE mice (control, *n* = 6; OE, *n* = 7) were fasted for 2 h, then they received a sucrose gavage at 2 g/kg. Sucrose-gavaged mice were killed after 1 h, blood was collected and serum was prepared. The level of mouse FGF21 proteins in each serum sample was determined with the Fibroblast Growth Factor 21 Mouse/Rat ELISA kit (BioVendor) according to the manufacturer’s instructions.

### Leptin level measurements

Eleven-week-old, male WT, OE-OS and OS-KO mice fed NC ad libitum (*n* = 8 per group) were killed, blood was collected and serum was prepared. The level of mouse leptin proteins in each serum sample was determined with the Mouse Leptin ELISA Kit (ab199082; Abcam) according to the manufacturer’s instructions.

### NAD^+^ measurements

We performed quantification of hypothalamic NAD^+^ levels in brain samples from 11-week-old male C57BL/6 mice, as previously described^10^ (*n* = 12 in the Fed group, and *n* = 13 in the Fasted group).

### Oxt release measurements

The measurements of Oxt release from ex vivo neurons of PVH nuclei from 11-week-old male C57BL/6 mice were performed as previously described^69^.

### Statistical analysis

The data were analysed with the Student’s *t*-test and Tukey’s multiple comparison test following one-way analysis of variance with SPSS 21.0J software. The data are expressed as the mean ± standard error (SE). All results with *p* values < 0.05 were considered statistically significant.

## Electronic supplementary material

Supplementary Information

## Data Availability

The data that support the findings of this study are available from the corresponding authors upon reasonable request.
