# Supplementary material for: Neuronal SIRT1 regulates macronutrient-based diet selection through FGF21 and oxytocin signalling in mice
Source: Nat Commun. 2018 Nov 2;9:4604. doi: 10.1038/s41467-018-07033-z (PMC6214990; doi:10.1038/s41467-018-07033-z)
Supplement: Supplementary file 1 — Supplementary Information [file 41467_2018_7033_MOESM1_ESM.pdf]

The Supplementary Information

Neuronal SIRT1 regulates macronutrient-based diet selection through FGF21 and oxytocin signalling in mice

Matsui et al.,

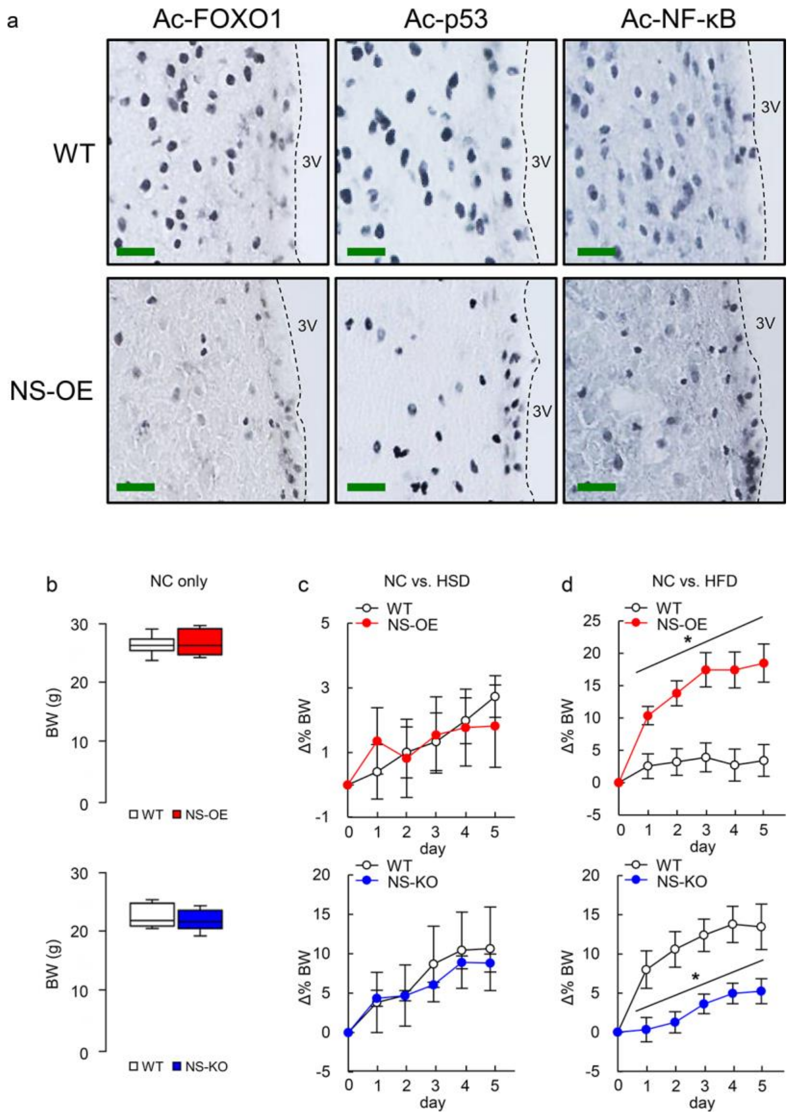

Supplementary figure 1

Effect of neuronal SIRT1 manipulation on the acetylation levels of SIRT1 substrates in the hypothalamus and body weight

(**a**) Photomicrographs depict hypothalamus coronal section from WT and NS-OE mice that received 3rd ICV injection of 0.5μl of TSA (10μg/μl). The length of scale bars is 100 μm. The sections were immunostained to identify Ac-FOXO1, Ac-p53, and Ac-NF-κB protein (black or blue) expression. (**b**) Body weight (BW) of mice under one-food access conditions, for neuron-specific SIRT1 overexpression (NS-OE, red) and neuron-specific SIRT1 knockout (NS-KO, blue) animals compared to wild-type mice (WT) (n=6 per group). (**c, d**) Percent changes in BW (Δ%BW) during the 5-day study: NC vs. HSD (**c**) and NC vs. HFD (**d**) (n=6 per group). Data in (**b**) are shown as box and whisker plots (center line, median; box limits, upper and lower quartiles; whiskers, the minimum and maximum value of a data set). Data in (**c**, **d**) represent mean±SE. *, p<0.05. Abbreviations: 3V, 3rd ventricle; Ac, acetylated.

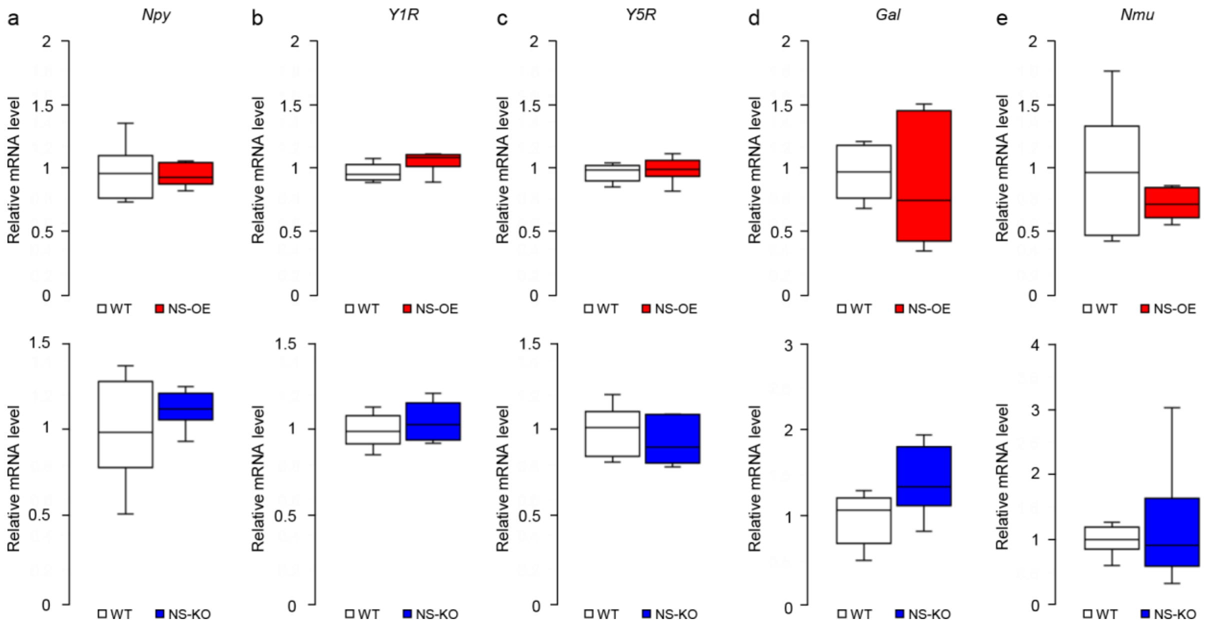

Supplementary figure 2

Neuronal SIRT1 does not alter expression of other genes that affect macronutrient-specific preferences

(**a**–**e**) Relative hypothalamic expression levels for NS-OE (red) and NS-KO (blue) mice: neuropeptide Y (*Npy*) (**a**), NPY receptors Y1 (*Y1R*) (**b**) and Y5 (*Y5R*) (**c**), galanin (*Gal*) (**d**), and neuromedin U (*Nmu*) (**e**) (n=6 per group). Data are shown as box and whisker plots (center line, median; box limits, upper and lower quartiles; whiskers, the minimum and maximum value of a data set).

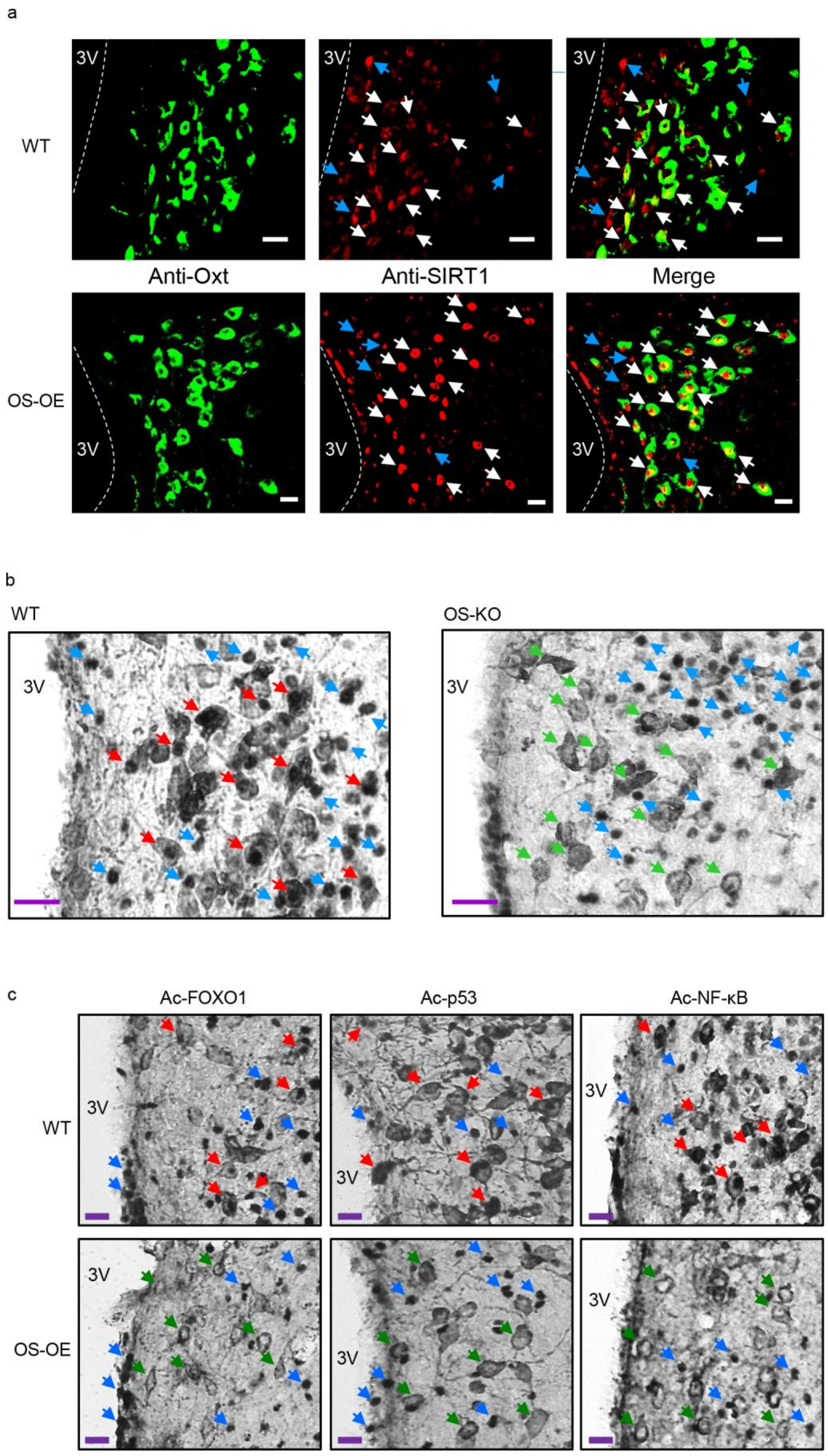
a
WT
OS-OE
3V
Anti-Oxt
Anti-SIRT1
Merge
b
WT
OS-KO
3V
c
Ac-FOXO1
Ac-p53
Ac-NF-κB
WT
OS-OE
3V

Supplementary figure 3

Immunohistological confirmation of Oxt neuron–specific SIRT1 manipulation

(**a**) Representative immunofluorescent images of coronal PVH sections of WT and OS-OE mice, immunostained for Oxt (green) and SIRT1 (red). White arrows indicate SIRT1 stains in Oxt (+) neurons. Blue arrows indicate SIRT1 stains in Oxt (-) neurons. (**b**) Photomicrographs depict coronal PVH sections from WT and OS-KO mice, immunostained for Oxt (grey) and SIRT1 (black). Red arrows indicate Oxt (+) neurons in WT mice, showing SIRT1 stains. Green arrows indicate Oxt (+) neurons in OS-KO mice, lacking SIRT1 stain. Blue arrows indicate Oxt (-) neurons with SIRT1 stains. The length of scale bar is 20 μm. (**c**) Photomicrographs depict hypothalamus coronal section from WT and OS-OE mice that received 3rd ICV injection of 0.5μl of TSA (10μg/μl). The length of scale bars are 100 μm. The sections were immunostained to identify Ac-FOXO1 (black), Ac-p53 (black), Ac-NF-κB (black) and Oxt (grey) protein expression. Blue arrows indicate Oxt (-)/Ac-FOXO1, Ac-p53, or Ac-NF-κB (+) neurons; red arrows indicate Oxt (+)/Ac-FOXO1, Ac-p53, or Ac-NF-κB (+) neurons; green arrows indicate Oxt (+)/Ac-FOXO1, Ac-p53, or Ac-NF-κB (-) neurons. Abbreviations: 3V, 3rd ventricle; Ac, acetylated.

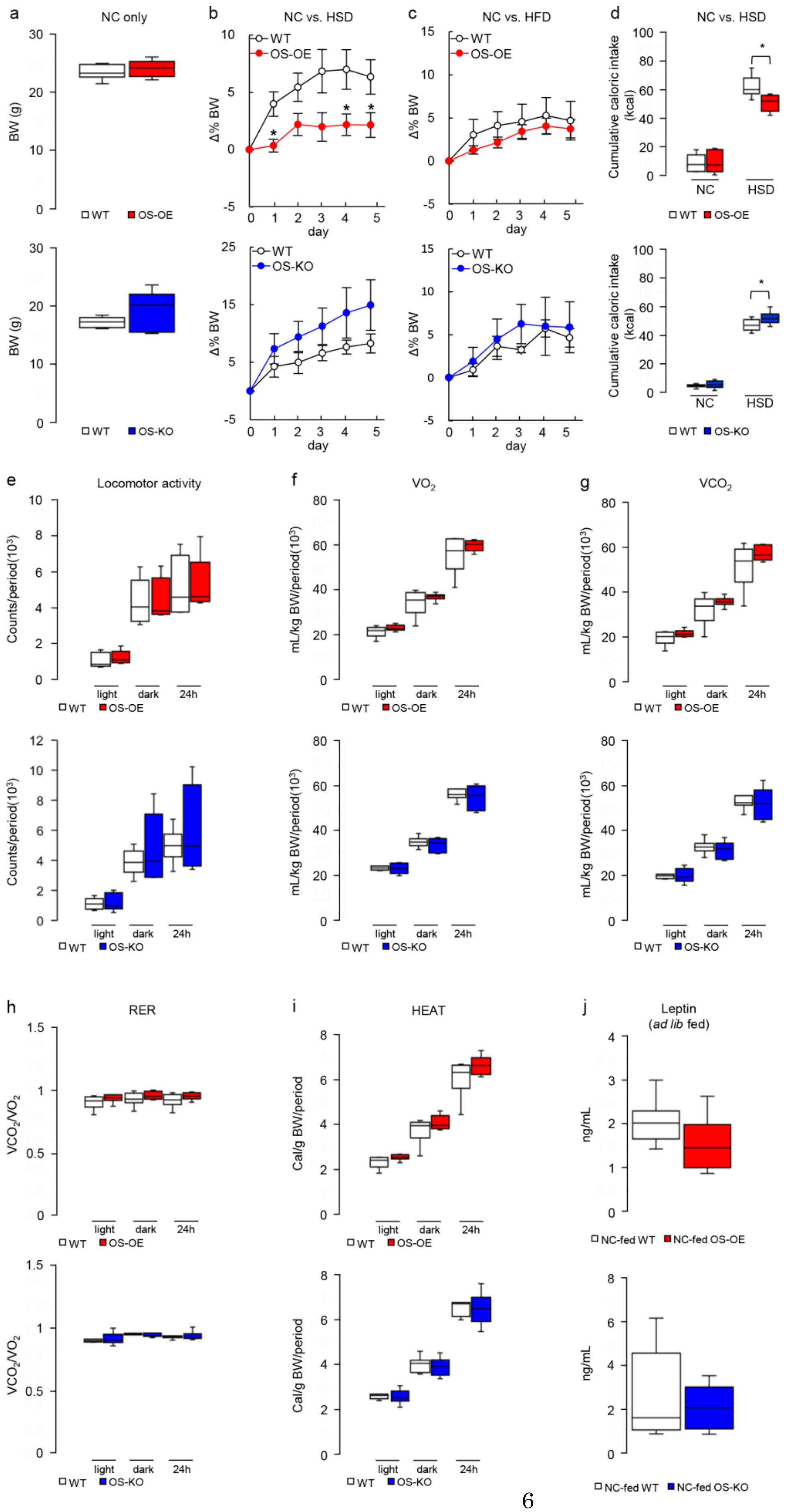
a
NC only
BW (g)
WT OS-OE
WT OS-KO
b
NC vs. HSD
WT
OS-OE
OS-KO
Δ% BW
day
c
NC vs. HFD
d
NC vs. HSD
Cumulative caloric intake (kcal)
NC
HSD
e
Locomotor activity
Counts/period(10^3)
light
dark
24h
f
VO_2
mL/kg BW/period(10^3)
g
VCO_2
h
RER
VCO_2/VO_2
i
HEAT
Cal/g BW/period
j
Leptin
(*ad lib* fed)
ng/mL
NC-fed WT NC-fed OS-OE
NC-fed WT NC-fed OS-KO

Supplementary figure 4

Effect of Oxt neuron–specific SIRT1 manipulation on body weight, locomotor activity, metabolic profiles, and plasma leptin level

(**a**) Body weight (BW) of mice under one-food access conditions, for Oxt neuron–specific SIRT1 overexpression (OS-OE, red) and Oxt neuron–specific SIRT1 knockout (OS-KO, blue) animals compared to wild-type (WT) (n=6 per group). (**b**, **c**) Percent changes in BW (Δ%BW) during the 5-day study: NC vs. HSD (**b**) and NC vs. HFD (**c**) (n=6 per group). (**d**) Cumulative caloric intake for two-choice diets in 5-day diet selection experiments (NC vs. HSD) in female OS-OE and OS-KO animals compared to wild-type mice (WT) (n=6 per group). (**e-i**) Locomotor activity (**e**), oxygen consumption (**f**), CO_2 production (**g**), respiratory exchange ratio (RER) (**h**), and heat generation (**i**) of male OS-OE mice (control, n=6; OE, n=7) and OS-KO mice (n=6 per group) at 8 weeks of age. (**j**) Serum leptin levels of *ad libitum* fed male OS-OE (n=8 per group) and OS-KO mice (n=8 per group). Data in (**b**, **c**) represent mean±SE. Data in (**a**, **d**, **e**, **f**, **g**, **h**, **i**, **j**) are shown as box and whisker plots (center line, median; box limits, upper and lower quartiles; whiskers, the minimum and maximum value of a data set). *, $p<0.05$.

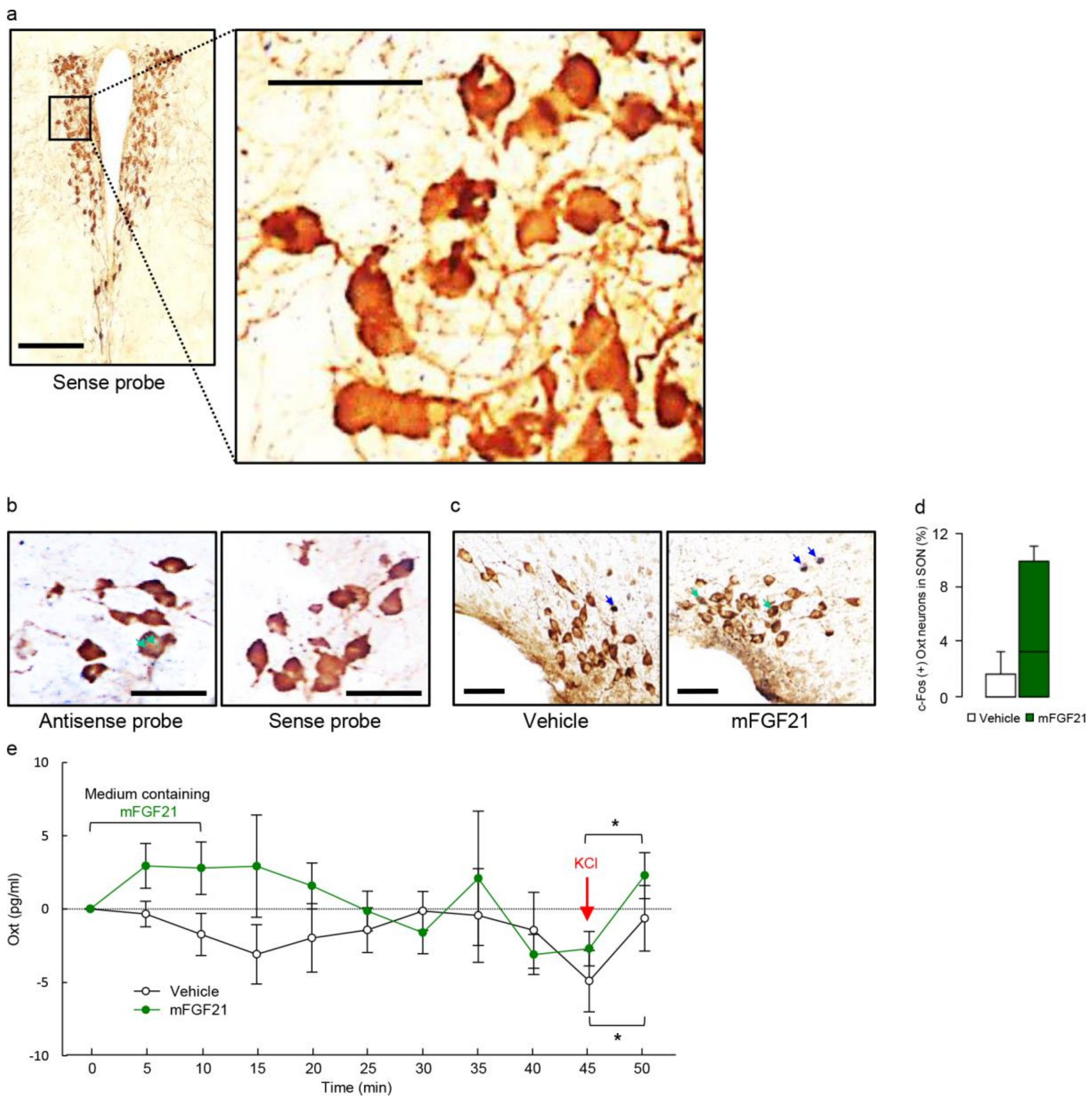

Supplementary figure 5

FGF21 does not stimulate somato-dendritic release of Oxt *ex vivo*

(**a-b**) Photomicrographs depict coronal PVH (**a**) and SON (**b**) sections from wild-type C57BL/6 mice. Control *in situ* hybridization was performed using *β-Klotho* sense mRNA probe for PVH (**a**), and antisense and sense mRNA probes for SON (**b**). The length of scale bar is 200 μm for the low magnification panel and 50 μm for the high magnification panel in (**a**); 50 μm in (**b**, **c**). Sections were immunostained to identify Oxt protein (brown) expression. (**c**) Photomicrographs depict coronal SON sections from wild-type C57BL/6 mice injected with murine FGF21 (mFGF21) (1 mg/kg, IP) or water (vehicle). The sections were immunostained for c-Fos (black) and Oxt (brown). Blue arrows indicate Oxt (-) c-Fos (+) neurons; green arrows indicate c-Fos (+) Oxt (+) neurons. (**d**) The percentage of c-Fos (+) cells among SON Oxt neurons after an IP injection of vehicle or mFGF21 (n=4 per group). **(e**) Time course of Oxt release from *ex vivo* PVH nuclei after addition of

100 nM mFGF21 (bracket) or vehicle. KCl (50 mM) was added at the end of the experiment to confirm sample viability (n=8 in the vehicle group, n=16 in the mFGF21 group). Data in (**d**) are shown as box and whisker plots (center line, median; box limits, upper and lower quartiles; whiskers, the minimum and maximum value of a data set). Data in (**e**) represent mean±SE. *, $p<0.05$.

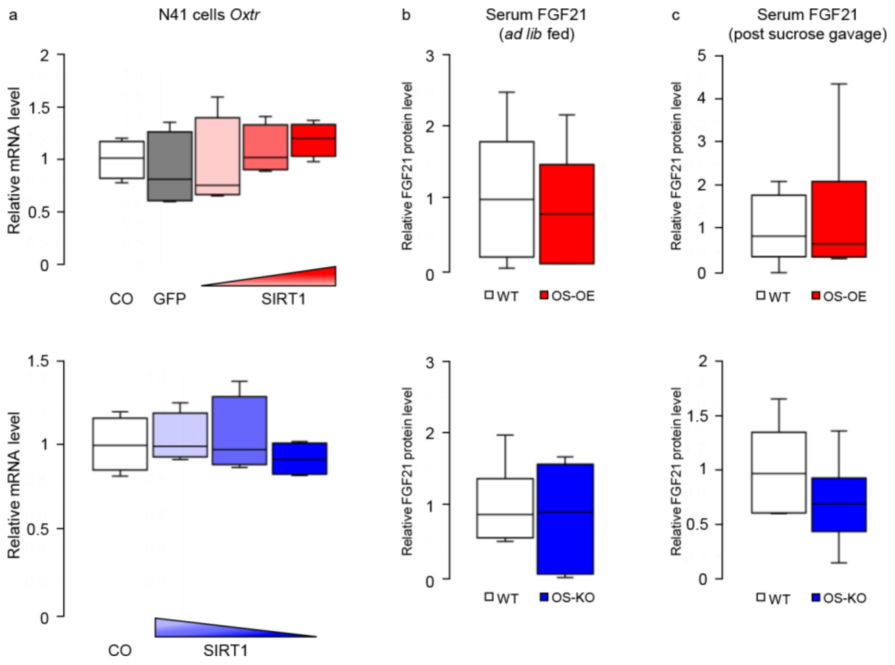

Supplementary figure 6

SIRT1 manipulation does not affect *Oxtr* expression *in vitro* and serum FGF21 levels *in vivo*

(**a**) The effects of overexpressing (*upper panels*) or silencing (*lower panels*) *Sirt1* expression in N41 cells on *Oxtr* mRNA expression (n=4 per group). (**b, c**) Serum FGF21 levels in OS-OE and OS-KO mice (in the *ad libitum* fed condition (**b**) (n=5 per group) ; 1 h after oral sucrose gavage in OS-KO mice (control, n=6; KO, n=6) and OS-OE mice (control, n=6; OE, n=7) (**c**). Data are shown as box and whisker plots (center line, median; box limits, upper and lower quartiles; whiskers, the minimum and maximum value of a data set). Data in (**a**) were replicated more than three times in the laboratory.

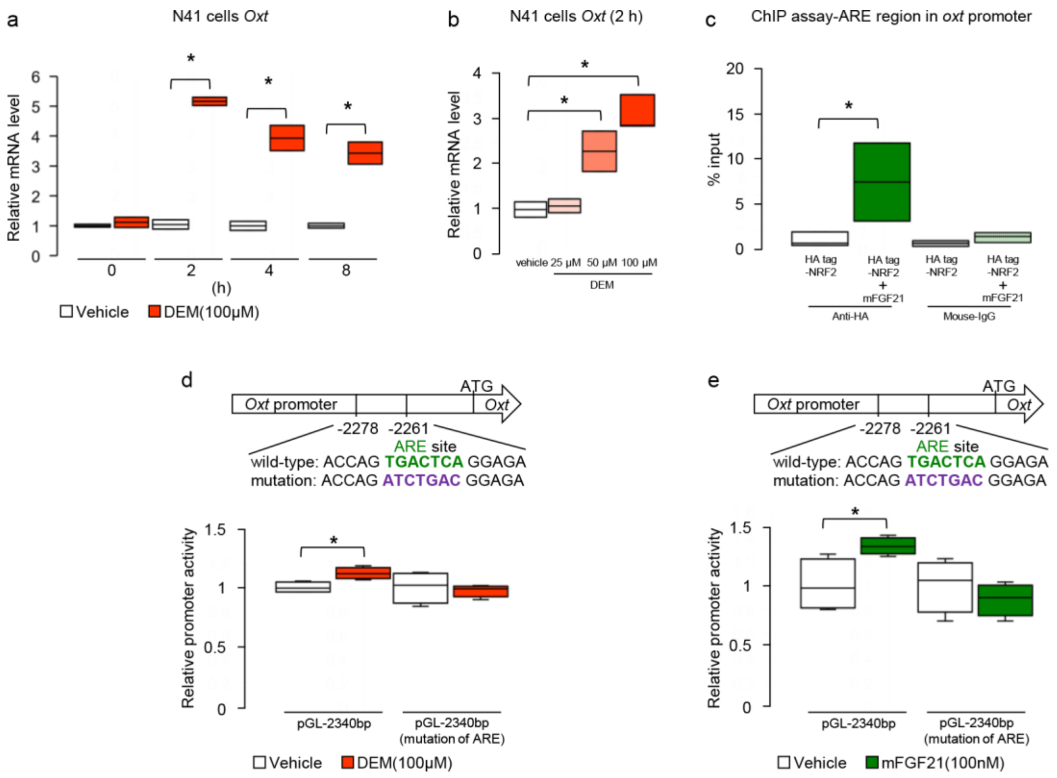

Supplementary figure 7

NRF2 mediates FGF21-induced *Oxt* expression, and NRF2 activation is sufficient for activating *Oxt* promoter

(**a, b**) The effects of activating NRF2 with diethyl maleate (DEM) on *Oxt* mRNA expression in N41 cells: time-course data (**a**) and dose-response data (**b**) (n=3 per group). (**c**) Chromatin immunoprecipitation (ChIP) assays performed on HA-tagged NRF2-transfected N41 cells with or without 100 nM mFGF21 treatment using HA tag or mouse IgG antibody. ChIP DNA was subjected to quantitative PCR using primers specific for the ARE region in *Oxt* promoter. Data are presented relative to input (n=3 per group). (**d, e**) Activity of the *Oxt* promoter with and without mutations in the ARE region; the effect of DEM (**d**) and 100 nM mFGF21 (**e**) (n=4 per group). Data are shown as box and whisker plots (center line, median; box limits, upper and lower quartiles; whiskers, the minimum and maximum value of a data set). *, $p<0.05$. Data in (**a, b**) were replicated more than three times, and data in (**c-e**) were replicated twice in the laboratory.

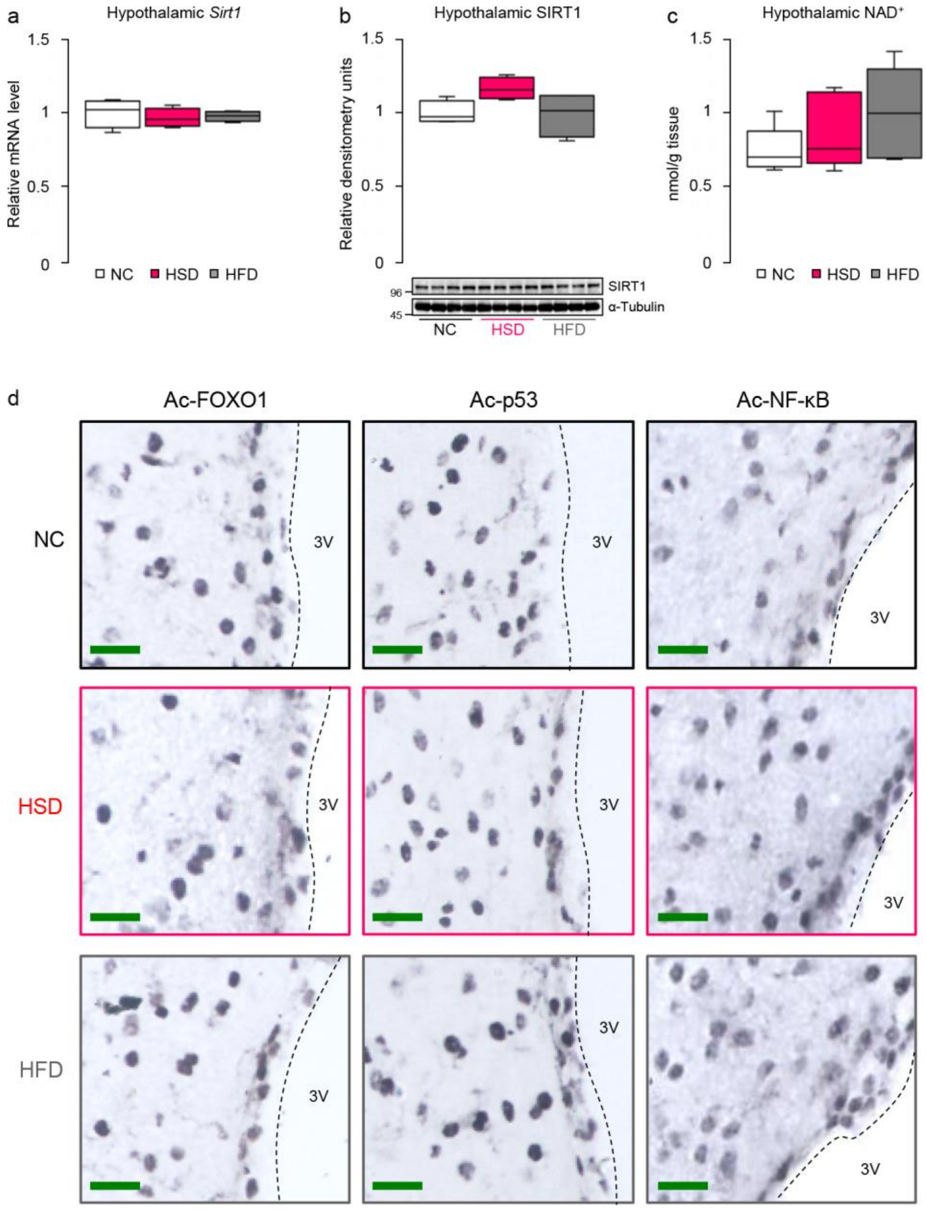

Supplementary figure 8

Effect of diet on hypothalamic SIRT1, NAD$^+$ and SIRT1 substrates acetylation levels

(**a–c**) Effects of only NC, HSD, or HFD diet for 3 days on hypothalamic *Sirt1* expression (**a**) (n=4 per group), SIRT1 protein levels (**b**) (n=4 per group), and NAD$^+$ levels (**c**) (n=6 per group) in WT C57BL6/J mice. Data are shown as box and whisker plots (center line, median; box limits, upper and lower quartiles; whiskers, the minimum and maximum value of a data set). (**d**) Photomicrographs depict hypothalamus coronal section from wild-type C57BL/6 mice (fed NC, HSD, or HFD) that received 3rd ICV injection of 0.5μl of TSA (10μg/μl). The length of scale bars are 100 μm. The sections were immunostained to identify Ac-FOXO1, Ac-p53, and Ac-NF-κB protein (black or blue) expression. Abbreviations: 3V, 3rd ventricle; Ac, acetylated.

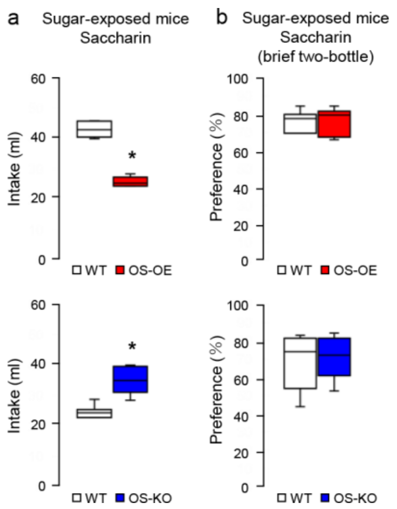

Supplementary figure 9

Sugar-exposed OS-OE and OS-KO mice showed altered saccharin preference

(**a, b**) Results of two-bottle choice tests with 0.2% saccharin over water, given for 3 days (**a**) (n=7 in the WT group; n=6 in the OS-OE and OS-KO groups), or for 10 min brief access (**b**) (n=6 per group). Data are shown as box and whisker plots (center line, median; box limits, upper and lower quartiles; whiskers, the minimum and maximum value of a data set). *, $p<0.05$.

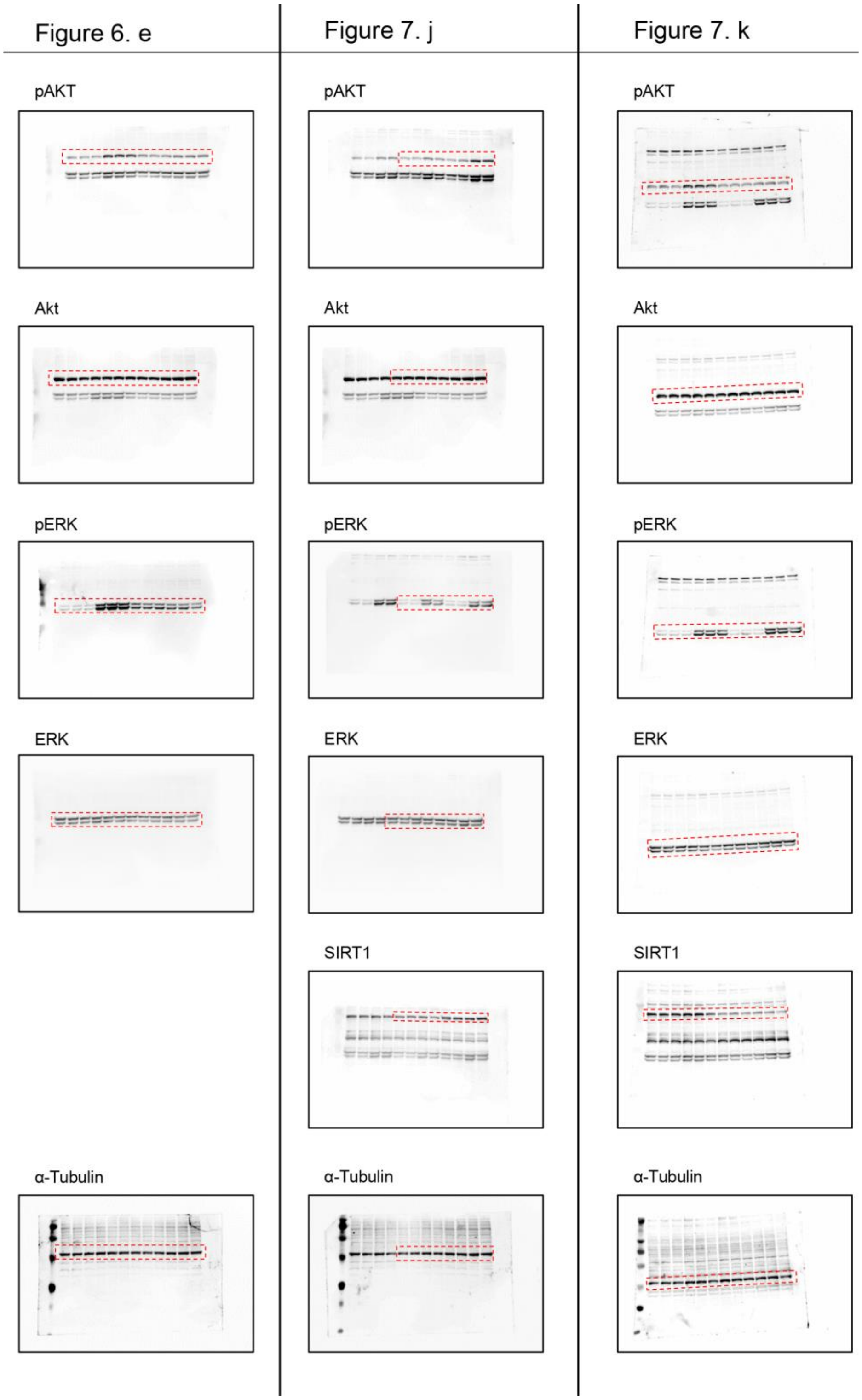

Supplementary figure 10

Full blots or gels for the data used in the figures

Full blots of Fig. 6e, 7j, and 7k.

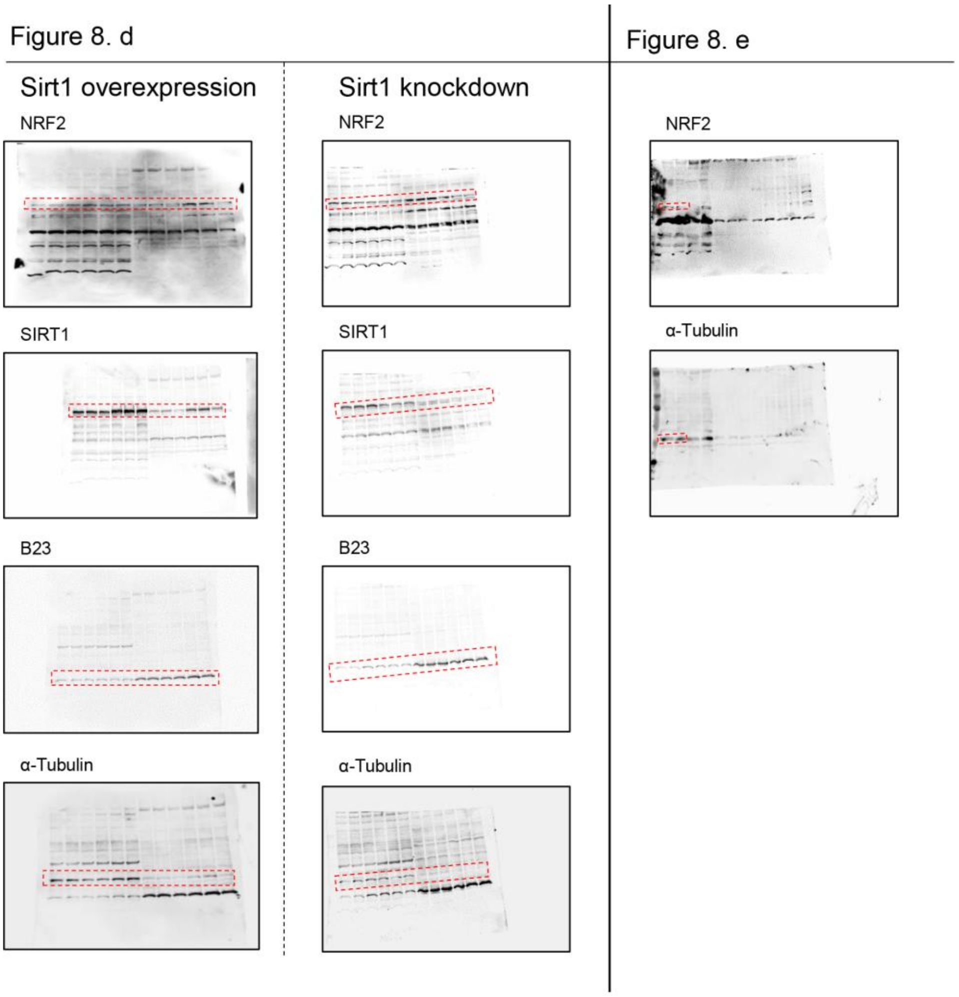

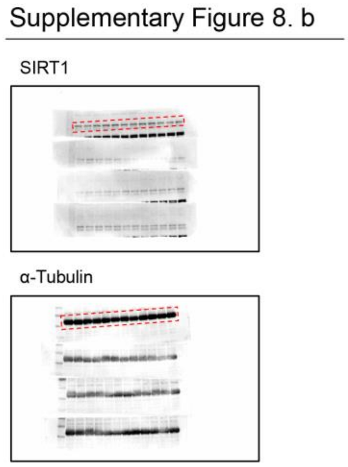

Supplementary figure 11

Full blots or gels for the data used in the figures

Full blots of Fig. 8d, 8e, and S8b.

Supplementary Table 1.

Primer sequences used for quantification of gene expression

Genes	Forward primer 5' to 3'	Reverse primer 3' to 5'
	Quantitative PCR	
Mouse *Sirt1*	GTAAGCGGCTTGAGGG	TTCGGGCCTCTCCGTA
Mouse *Sirt1* (*Exon4*)	GATGCTGTGAAGTTACTGCAGGAGTG	GAGGGTCTGGGAGGTCTGGGAAG
Mouse *Oxt*	TGGCTTACTGGCTCTGACCT	AGGCAGGTAGTTCTCCTCCTG
Mouse *Otr*	GGAGCGTCTGGGACGTCAAT	AGGAAGCGCTGCACGAGTT
Mouse *Klb*	GTTCTGCTGCGAGCTGTTAC	TACCGGACTCACGTACTGTTT
Mouse *Fgfr1*	TGTTCTGCTGCGAGCTGTTAC	TACCGGACTCACGTACTGTTT
Mouse *Npy*	TACTCCGCTCTGCGACACTA	TCTTCAAGCCTTGTTCTGGG
Mouse *Npy Y1r*	CAAGATATACATTCGCTTGA	AGATTGTGGTTGCAGG
Mouse *Npy Y5r*	TCAAGCGTTCCCTCAC	ACAACAGGACATCATGC
Mouse *Gal*	CAGCCTTGATCCTGCACTGA	CAGGGTCACAACCAACAGGA
Mouse *Nmu*	CTGAGGGAGCTTTGCCGTAT	CAACGGATGCACAACAGAGG
Mouse *β-actin*	AGCCTTCCTTCTTGGGTA	GAGCAATGATCTTGATCTTC
	Chromatin immunoprecipitation	
ARE region in Oxt promoter	TCACCACCCACTTAGCGTTG	AACCTTCTCCTCAAGGGGCT

Supplementary Table 2.

Primer sequences used to generate *in situ* hybridization probes

Gene	Forward primer 5' to 3'	Reverse primer 3' to 5'
Mouse *Klb*	GGGTCTCCGGGGAATGAATG	TTGGGTTTACCGGACTCACG
